# Ultrastructural and molecular analysis of the origin and differentiation of cells mediating brittle star skeletal regeneration

**DOI:** 10.1186/s12915-020-00937-7

**Published:** 2021-01-18

**Authors:** Laura Piovani, Anna Czarkwiani, Cinzia Ferrario, Michela Sugni, Paola Oliveri

**Affiliations:** 1 Department of Environmental Science and Policy, University of Milan, Via Celoria, 2, 20133 Milan, Italy; 2 Department of Genetics, Evolution and Environment, University College London, London, UK; 3 Center for Life Origins and Evolution, University College London, London, UK; 4 Present Address: DFG-Center for Regenerative Therapies Technische Universität Dresden (CRTD), Dresden, Germany; 5 Center for Complexity and Biosystems, Department of Physics, University of Milan, Via Celoria, 16, 20133 Milan, Italy; 6 GAIA 2050 Center, Department of Environmental Science and Policy, University of Milan, Via Celoria, 2, 20133 Milan, Italy

**Keywords:** Regeneration, Echinodermata, *Amphiura filiformis*, Osteogenesis, Cell differentiation, Biomineralization, Gene expression

## Abstract

**Background:**

Regeneration is the ability to re-grow body parts or tissues after trauma, and it is widespread across metazoans. Cells involved in regeneration can arise from a pool of undifferentiated proliferative cells or be recruited from pre-existing differentiated tissues. Both mechanisms have been described in different phyla; however, the cellular and molecular mechanisms employed by different animals to restore lost tissues as well as the source of cells involved in regeneration remain largely unknown. Echinoderms are a clade of deuterostome invertebrates that show striking larval and adult regenerative abilities in all extant classes. Here, we use the brittle star *Amphiura filiformis* to investigate the origin and differentiation of cells involved in skeletal regeneration using a combination of microscopy techniques and molecular markers.

**Results:**

Our ultrastructural analyses at different regenerative stages identify a population of morphologically undifferentiated cells which appear in close contact with the proliferating epithelium of the regenerating aboral coelomic cavity. These cells express skeletogenic marker genes, such as the transcription factor *alx1* and the differentiation genes *c-lectin* and *msp130L*, and display a gradient of morphological differentiation from the aboral coelomic cavity towards the epidermis. Cells closer to the epidermis, which are in contact with developing spicules, have the morphology of mature skeletal cells (sclerocytes), and express several skeletogenic transcription factors and differentiation genes. Moreover, as regeneration progresses, sclerocytes show a different combinatorial expression of genes in various skeletal elements.

**Conclusions:**

We hypothesize that sclerocyte precursors originate from the epithelium of the proliferating aboral coelomic cavity. As these cells migrate towards the epidermis, they differentiate and start secreting spicules. Moreover, our study shows that molecular and cellular processes involved in skeletal regeneration resemble those used during skeletal development, hinting at a possible conservation of developmental programmes during adult regeneration. Finally, we highlight that many genes involved in echinoderm skeletogenesis also play a role in vertebrate skeleton formation, suggesting a possible common origin of the deuterostome endoskeleton pathway.

## Background

Regeneration is the ability to re-grow lost body parts after trauma. After an initial phase of wound healing, new cells are produced, specified, and finally reorganized into functional tissues and complex structures.

Among metazoans, the ability to regenerate is highly variable. Some animals can regenerate their entire body from small fragments, such as cnidarians or flatworms, whereas others can restore only a few tissues or organs, like some vertebrates [1–4]. Not only does the extent of regenerative ability vary among metazoans, different animals employ diverse cellular mechanisms to regenerate lost body parts. New cells can originate from proliferation of pre-existing undifferentiated cells (such as stem cells) or from remodelling of stump differentiated tissues. The origin of cells involved in regeneration and the molecular processes underlying their differentiation remain hotly debated topics [2, 5, 6].

Investigating the cellular and molecular mechanisms involved in regeneration of different phyla can help understand the evolutionary origin of this phenomenon [4, 7, 8]. Among deuterostomes, echinoderms show the most striking regenerative capacities. In fact, both adults and planktonic larvae of all five extant classes can extensively regenerate [5, 9–12].

Comparative morphological studies have shown that echinoderms use different types of regenerative strategies that rely either on cells of existing tissues, which dedifferentiate and then re-differentiate, or on proliferating stem cells [8, 11, 13, 14]. Besides regeneration, another striking feature of echinoderms is their mesoderm-derived endoskeleton, which is composed of a three-dimensional meshwork, called stereom, made of magnesium-rich calcite deposited on an organic matrix [15, 16]. Ultrastructural studies of non-regenerating skeleton show that sclerocytes (cells responsible for building the skeletal structures) are characterized by mononucleated and roundish cell bodies anchored to the calcite trabeculae by stalks and are surrounded by a secondary boundary layer [17–19].

Most of what is known about cellular and molecular mechanisms of skeleton formation in echinoderms comes from embryonic data from sea urchins and, more recently, from the brittle star *Amphiura filiformis* [20–25]. In both sea urchins and brittle stars, the skeleton is secreted by a population of mesodermal cells that detaches from the vegetal pole of the embryo and undergoes an epithelial-to-mesenchymal transition (EMT) [20]. The developmental programme from early cell specification to final differentiation has been revealed by systems-level studies and genome-wide analysis of gene regulatory networks (GRNs). These studies identified several transcription factors (TFs) responsible for the specification of cell identity and the activation of a large set of terminal differentiation genes, such as skeletogenic matrix (SM) genes, *p19*, *c-lectin*, *msp130*, collagens, and many more [23–28]. Many of these genes show conserved expression in the sclerocytes of both sea urchin and brittle star embryos [24].

As for adult skeletal regeneration, most of the work carried out so far has focused on spine or test regeneration in sea urchins, and arm regeneration in starfish [29–31] and the brittle star *A. filiformis* [32–34]. This burrowing brittle star is ideal for studying cellular and molecular aspects of development and regeneration due to its small size, fast regeneration, almost transparent regenerating arms, and accessible annotated transcriptome [24, 25, 34–37].

**Fig. 1:**
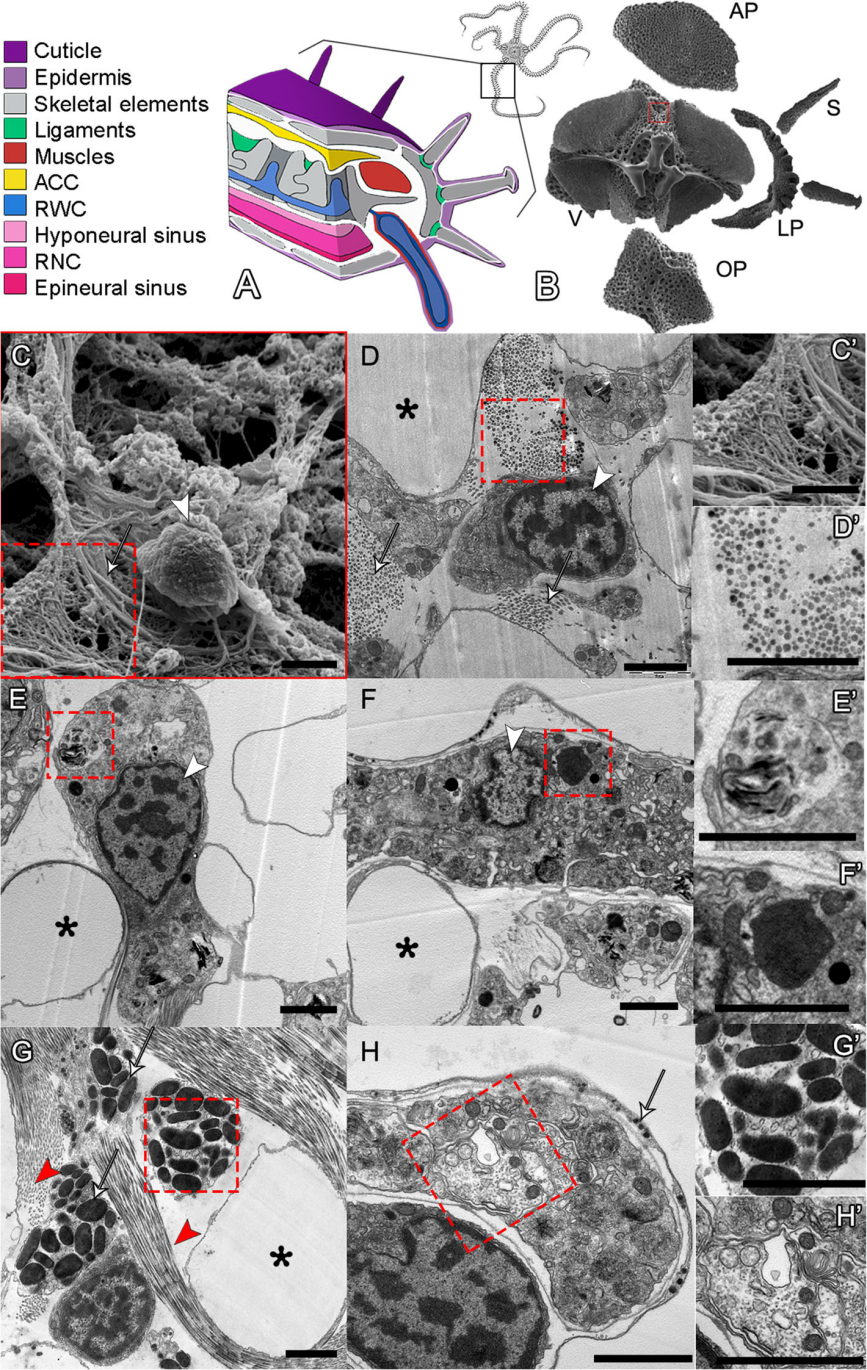
*A. filiformis* non-regenerating arm skeletal elements and ultrastructure of cell types. **A** 3D schematic representation of a non-regenerating arm showing the main anatomical structures colour coded as in legend on the left. **B** SEM of the skeletal elements present in a single metameric unit and displayed in their corresponding anatomical position. AP, aboral arm plate; LP, lateral arm plate; OP, oral arm plate; S, spine; V, vertebra. **C** SEM micrograph of a cell in the stroma of the skeleton of the vertebra: arrowhead indicates the nucleus, white arrow points at fibrils with morphology typical of collagen, magnified in **C’**. **D** TEM micrograph of a sclerocyte in the vertebra: white arrows indicate fibrils in cross section likely of collagen, magnified in **D’**. **E** TEM micrograph of a presumptive pigment cell in the oral arm plate with an evident nucleolus and spindle-shaped electron-dense structures typical of pigment granules, magnified in **E’**. **F** TEM micrograph of a phagocyte in the aboral arm plate characterized by the presence of phagosome, magnified in **F’**. **G** TEM micrograph of a granulocyte present in the vertebra: red arrowheads highlight fibrils, present in both cross and longitudinal sections; arrows point at large electron-dense granules of different shapes and sizes, magnified in **G’**. **H** TEM micrograph of a nerve cell in the aboral arm plate characterized by a nerve process magnified in **H’**; white arrow indicates the small electron-dense roundish vesicles generally containing neurotransmitters or other neuro-signalling molecules. Scale bars = 2 μm. In all images: asterisks indicate the presence of biomineralized skeletal tissue (stereom calcareous elements or trabeculae) now empty, i.e. electron-transparent due to fixation and decalcification processes; white arrowhead shows the nucleus with hetero- and euchromatin; and red dotted box indicates magnified part of the figure

Like other brittle stars, adults of *A. filiformis* possess a central disc and five segmented arms with three main continuous axial structures running along them (see Fig. 1A). These axial structures are from the aboral to the oral side: the aboral coelomic cavity (ACC; yellow in Fig. 1A), the radial water canal (RWC; blue in Fig. 1A), and the radial nerve cord (RNC; pink in Fig. 1A). In each segment, there is a set of five different skeletal elements (see Fig. 1B), namely a pair of lateral arm plates, an aboral and an oral arm plate, a central vertebra and a number of spines, four muscle bundles (red in Fig. 1A), ligaments (green in Fig. 1A), and a pair of podia (or tube feet), which are finger-like lateral extensions of the RWC surrounded by muscles, nervous system, and epidermis (from the inner to the outer layer).

The early stages of arm regeneration in *A. filiformis* have been divided by Czarkwiani and co-workers [34] into a five-stage process which begins with the closure of the wound (stage 1) and is followed by the formation of a regenerative bud (stage 2). It has been shown that this bud consists of five different regenerating tissues: the epidermis, the developing dermal layer, the ACC, the RWC, and the RNC. The emerging axial structures, however, become visible in whole mount only at around stage 3. At stage 4, the first newly formed segment becomes visible, and at stage 5, several segments are detectable. The most proximal segment, closest to the amputation plane, is the first segment formed and the most developed, while the most distal, closest to the proliferative zone, is the last segment formed and the least differentiated. After stage 5, the terminal tip of each regenerating arm becomes fully differentiated as shown by the presence of the terminal podium and the terminal ossicle. Proximally to this differentiated structure, a highly proliferative zone produces new segments, which intercalate between the stump and this terminal element in a proximal-distal orientation (i.e. the proximal segments are more developed than the distal ones) [34]. This is consistent with the process of regeneration by distalization-intercalation where the distal part of the body, or limb/arm, is regenerated first and gives positional information to start restoring original structures [38]. Previous studies have shown that around stage 3 the primordia of new ossicles, called spicules, appear in the developing dermal layer and that the cells of this area express skeletogenic markers, such as *alx1*, *ets1/2*, *gataC*, *c-lectin*, *p19*, and *p58b* [33, 34]. Cell proliferation assays using 5-ethynyl-2′-deoxyuridine (EdU) showed that the cells of this layer are not proliferating and that even at later stages of regeneration sclerocytes marked by differentiation markers do not proliferate [34]. Since the number of sclerocytes increases considerably during regeneration, but they do not proliferate, the origin of these cells remains unknown.

This work aims to understand the source of sclerocytes during arm regeneration using microscopy at different regenerative stages combined with molecular markers. In order to do this, we first identified various cell types present in the stroma of the fully differentiated skeleton of non-regenerating arms to assess their appearance and differentiation during regeneration. We then characterized regenerating cells at both ultrastructural and molecular level using a combination of new and known skeletogenic genes. Finally, we analysed the spatial expression of 19 genes at early and advanced regenerative stages to unravel the mechanisms used to re-establish the complexity of the skeletal elements in the brittle star arms.

Our data support the hypothesis that sclerocytes differentiate from a population of progenitor cells emerging from the epithelium of the aboral coelomic cavity. Furthermore, we show that different molecular signatures characterize the development of the different skeletal elements (e.g. vertebrae and arm plates).

## Results

### Histological and ultrastructural characterization of sclerocyte precursors

To better characterize cells involved in the regeneration of the skeleton in *A. filiformis*, we first performed histological and ultrastructural analyses of non-regenerating arms to identify mature cell types. This is because fully developed non-regenerating arms represent the end point of the regenerative process and the morphology of differentiated cells can be clearly distinguished. Histological and ultrastructural analyses were performed on the five skeletal elements at different positions in the arm. As shown in Fig. 1, we observed a variety of cell morphologies typical of sclerocytes (C, D); presumptive pigment cells (E), mainly found in the aboral and oral arm plates; phagocytes (F); granule cells (G); and neuronal cells (H). Among these cell types, sclerocytes represent the minority of the populations: they are characterized by a roundish cell body with little cytoplasm and a patchy nucleus, and are immersed in a matrix rich in fibrils and microfibrils, likely collagen (Fig. 1C, D). In fact, both the fibril diameters (visible in cross section) and D-period (visible in longitudinal section) are compatible with those of collagen fibrils.

We then studied the cell types present in the regenerating arms at different stages using similar structural and ultrastructural approaches. Consistent with previous studies, our analysis shows that the regenerative bud (stages 2 and 3) is composed of the covering epidermis (shown in purple in most schematics) and an inner bulk made by the regenerating axial structures, i.e. the ACC (yellow), the RWC (blue), and the RNC (pink) [34]. Between the epidermis and the axial structures, the dermal layer, where skeletal elements appear, is also present [34].

**Fig. 2:**
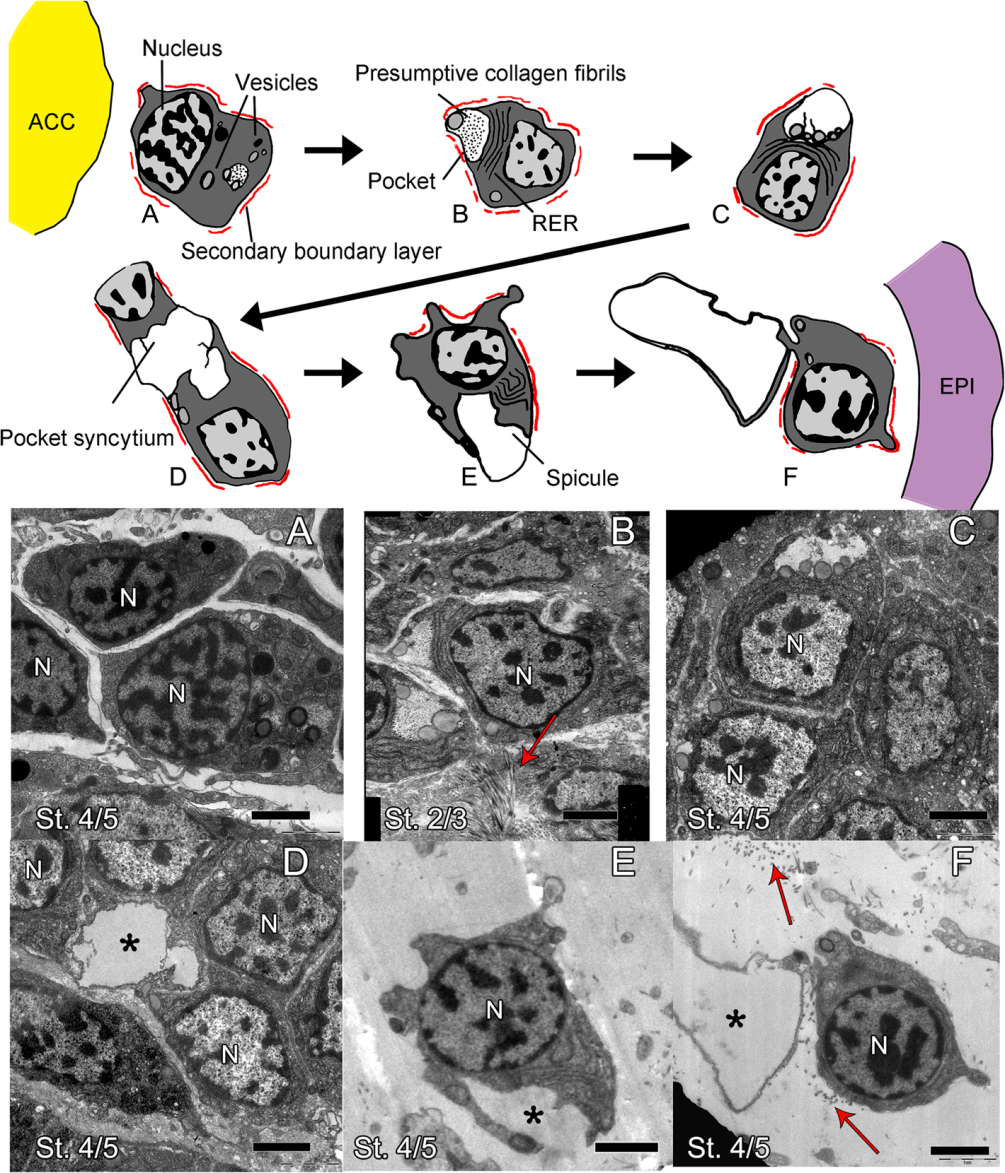
The main cell population of the dermis at early regenerative stages shows a gradient of cellular differentiation. Schematics (top) and TEM micrographs (**a**–**f**). **a** Mesenchymal cells at stage 4/5 located at the distal tip near the axial structures look rather undifferentiated. Main features are a secondary boundary layer (always highlighted in red in the schematics), a few vesicles, and a patchy nucleus. **b** Mesenchymal cells at stage 2/3 in the area next to where the ACC and RNC are adjacent on either side of the RWC. Cells show large RER, many vesicles, and a cytoplasmic pocket containing fibrils likely of collagen. **c** Mesenchymal cells at stage 4/5 at the very distal tip of the regenerate. Cells show large RER, vesicles, and a more electron-transparent cytoplasmic pocket. **d** Mesenchymal cells at stage 4/5 at the distal-most tip of the regenerate show a pocket syncytium. **e**, **f** Mesenchymal cells at stage 4/5 right under the epidermis show growing spicules. The main cellular features are indicated in the schematic the first time they appear. Red arrows indicate collagen fibrils, capital N indicates nucleus, and asterisks indicate growing spicules. ACC, aboral coelomic cavity; EPI, epidermis; St., stage. Scale bars = 2 μm

In the regenerating dermal layer, we observed only three distinct cell morphologies. Two out of these three were observed more rarely, and only from stage 3 onwards, and present the morphology of morula (or spherule) cells (Additional file 1 Fig. S1 A) and phagocytes (Additional file 1 Fig. S1 B). The more abundant cell population is characterized by a population of mesenchymal cells with highly patchy and heterochromatin-rich nuclei, abundant rough endoplasmic reticulum (RER) and mitochondria, electron-dense vesicles, and a secondary boundary layer (Fig. 2). Among cells of this population, we can observe some gradual differences between cells closer to the axial structures (ACC, RWC, and RNC) and those closer to the epidermis (Fig. 2 and Additional file 1 Fig. S2). Cells closer to the axial structures (Fig. 2a, b and Additional file 1 Fig. S2 A-C) appear very tightly packed and rather undifferentiated; they are overall roundish and have little cytoplasm and few cell inclusions. At all stages, these cells are localized where the ACC and the RNC meet at either side or in front of the RWC in the most distal part of the regenerative bud (see Additional file 1 Fig. S2). At later regenerative stages (from around stage 4 onwards), we can also find them in the space between the RWC and the ACC. Here, we can observe numerous cells containing phagosomes (Additional file 1 Fig. S2 C, red triangle) and apoptotic nuclei, characterized by condensation of chromatin on the nuclear membrane (Additional File 1 Fig. S2 C, red circle), signs of tissue remodelling that are scarcely detectable elsewhere. This is the area where later in the regeneration process (from stage 5) vertebral primordia will start to form [34] (see Additional file 1 Fig. S3).

**Fig. 3:**
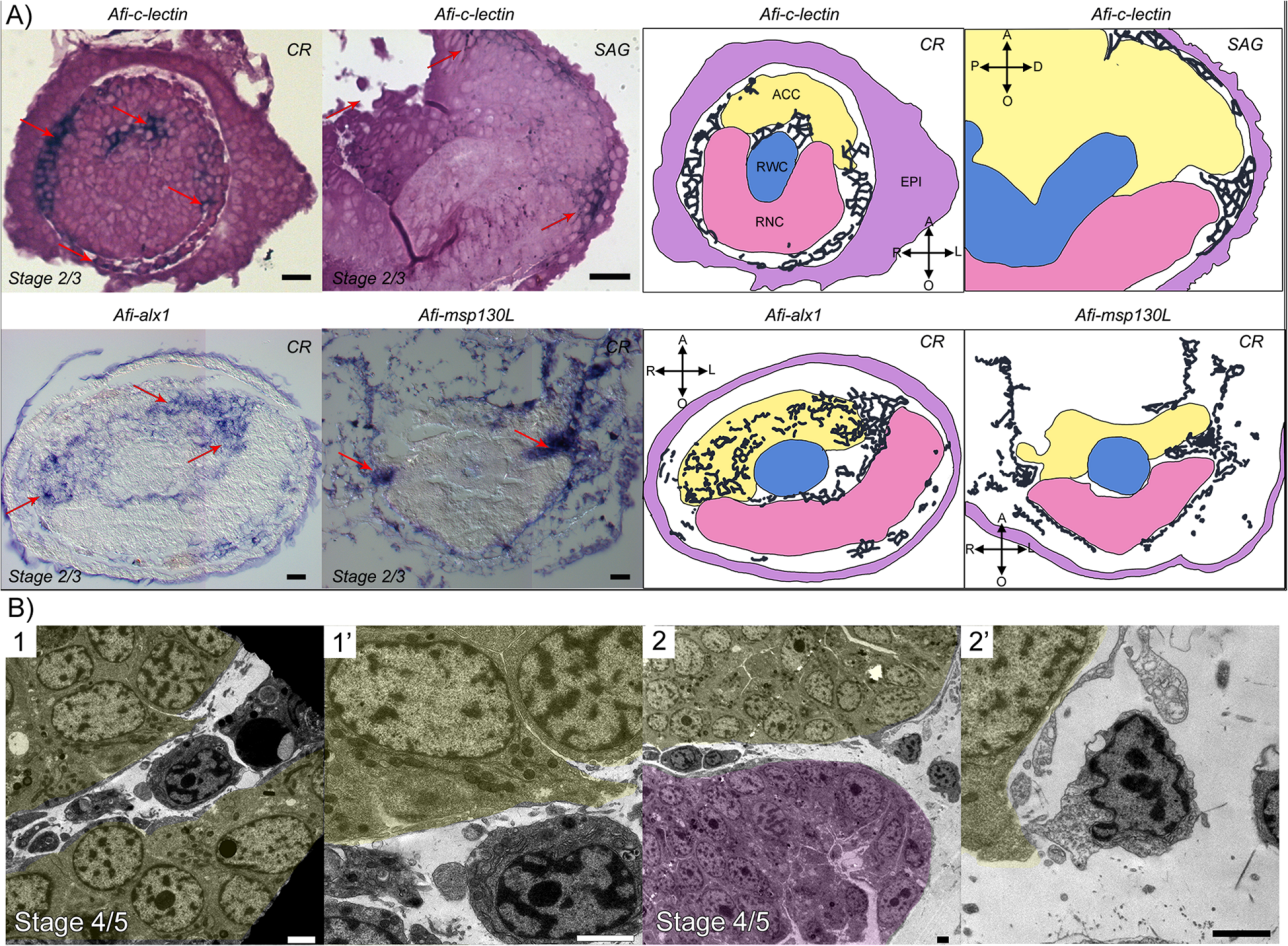
Regenerating mesenchymal cells express skeletogenic gene markers and appear to detach from the ACC epithelium. **a** Semi-thin sections of whole mount ISH for skeletal genes: *Afi-c-lectin* (embedded in resin), *Afi-alx1* and *Afi-msp130L* (embedded in wax). Red arrows point at areas where gene expression (dark blue/purple staining) is present, as schematized on the right. Yellow, aboral coelomic cavity; blue, radial water canal; pink, radial nerve cord; CR, cross section; SAG, sagittal section. Crossed arrows further indicate the orientation of the section: A, aboral; O, oral; R, right; L, left; P, proximal; D, distal. Scale bars = 10 μm. **b** TEM micrographs of cells likely detaching from the ACC epithelium. (1’) and (2’) are details of (1) and (2), respectively. Yellow, ACC; pink, RNC. Scale bars = 2 μm

In all areas described above, cells in the developing dermal (or mesenchymal) layer are in very close contact with the epithelium of the ACC, the RWC, and the RNC. However, they have a very different morphology from nearby cells of the axial structures which have a bigger, more electron-dense cytoplasm, large nucleolus, and many cell inclusions (compare mesenchymal cells in Fig. 2 and Additional file 1 Fig. A2 with Additional file 1 Fig. S4 A”, C, E and G). Cell boundaries within the axial structures are difficult to detect (see Additional file 1 Fig. S4 A”, C, E, G); however, the RWC (Additional file 1 Fig. S4 D, see arrow), the RNC (Additional file 1 Fig. S4 F, see arrow), and the epidermis (Additional file 1 Fig. S4 G’) show a clear basal lamina while the ACC apparently does not (Fig. S4 B). The epithelium lining of the ACC loses its continuity and “disaggregates”, especially on its oral side, facing the RWC and RNC (see Additional file 1 Fig. S4 A, A’ and A” and compare with Additional file 1 Fig. S4 C and E). Here, serial sections from different samples at different stages seem to show a few cells detaching from the ACC epithelium towards the mesenchymal space (Fig. 3b). Cells closer to the epidermis display gradually more cytoplasmic projections, often have a secondary boundary layer resembling those described for sclerocytes by Märkel and Röser [17], and display a pocket-like structure containing presumptive collagen fibrils. They also have a growing number of electron-opaque vesicles gathering next to this plasma membrane invagination (Fig. 2a–d). The content of the pocket of cells near the axial structures appears more electron-opaque than that of cells closer to the epidermis (compare Fig. 2b with Fig. 2c, d) possibly indicating that the composition of the pocket content changes. The short cell projections delimiting the pocket of one single cell never fuse together; therefore, this space remains “extracellular”. However, in some cases, different neighbouring cells “fuse” their projections to form a syncytium delimitating a shared extracellular space, i.e. pocket space (Fig. 2d). Cells immediately under the epidermis are scattered in a relatively abundant matrix, composed of banded collagen fibrils and microfibrils. Several sections show that these cells are often in very close proximity to developing spicules (Fig. 2e, f and Additional file 1 Fig. S2 D), and ultrastructural analysis shows a morphology similar to that of mature sclerocytes (compare Fig. 1D with Fig. 2e, f).

We observed that, morphologically, this population of mesenchymal cells adjacent to the epidermis appears more differentiated (i.e. they have a lower nucleus/cytoplasm ratio, several cytoplasmic projections, and various inclusions) closer to the amputation plane compared to the tip as well as in later stages of development (stage 4/5) compared to early ones (stage 2/3) (see the top of Additional file 1 Fig. S2). All cell morphologies described above have been consistently observed across different samples and stages unless otherwise stated in the text.

To summarize, by analysing serial sections of samples at different regenerative stages, we observed a population of mesenchymal cells, which appears to bud off from the oral side of the ACC that faces the RWC/RNC. These cells show a gradient of morphologies with cells closer to the axial structures having a more undifferentiated morphology than those closer to the epidermis. Cells right under the epidermis are often in close contact with developing spicules and resemble mature sclerocytes. Gene expression of molecular markers might help elucidate their true identity.

### Localization of skeletogenic gene expression at early stages of regeneration

To assess the identity of the cells described above, we carried out a large-scale gene expression study using in situ hybridization (ISH) of both transcription factors (TFs) and differentiation genes known to be involved in skeletogenesis. All ISH experiments were conducted and analysed in at least three replicates for each stage, and only consistent results are reported here. Whole mount samples were oriented for imaging, and both oral and aboral sides were analysed to determine the expression in ACC and RNC.

Firstly, we analysed the expression of the three skeletogenic markers *msp130-L*, *c-lectin*, and *alx1*; the latter two were previously shown to be broadly expressed in the dermal layer of *A. filiformis* regenerating arms [33, 34]. To gain better cellular resolution, we performed whole mount ISH and then sectioned the arms after embedding in wax and/or resin. Our sections show that not only cells under the epidermis but also those in close contact with axial structures and those present between the ACC and RWC at later stages show expression of these molecular markers (Fig. 3, red arrows). Moreover, our sections clearly show that *Afi-alx1* is also expressed in the oral side of the ACC epithelium (Fig. 3a).

To further characterize the molecular signature of these cells, we then selected 16 genes with known skeletogenic roles. Among these genes, we chose ten TFs of which six have a role in the sea urchin and/or brittle star embryonic skeletogenic gene regulatory networks (GRNs) (*erg*, *foxN2/3*, *jun*, *nk7*, *snail*, and *twist*) [23, 24, 26, 39–42]. We additionally cloned four TFs known to be involved in bone formation in vertebrates (*pax1/9*, *soxE*, *sp5*, and *sp7/8*) [43–48].

**Fig. 4:**
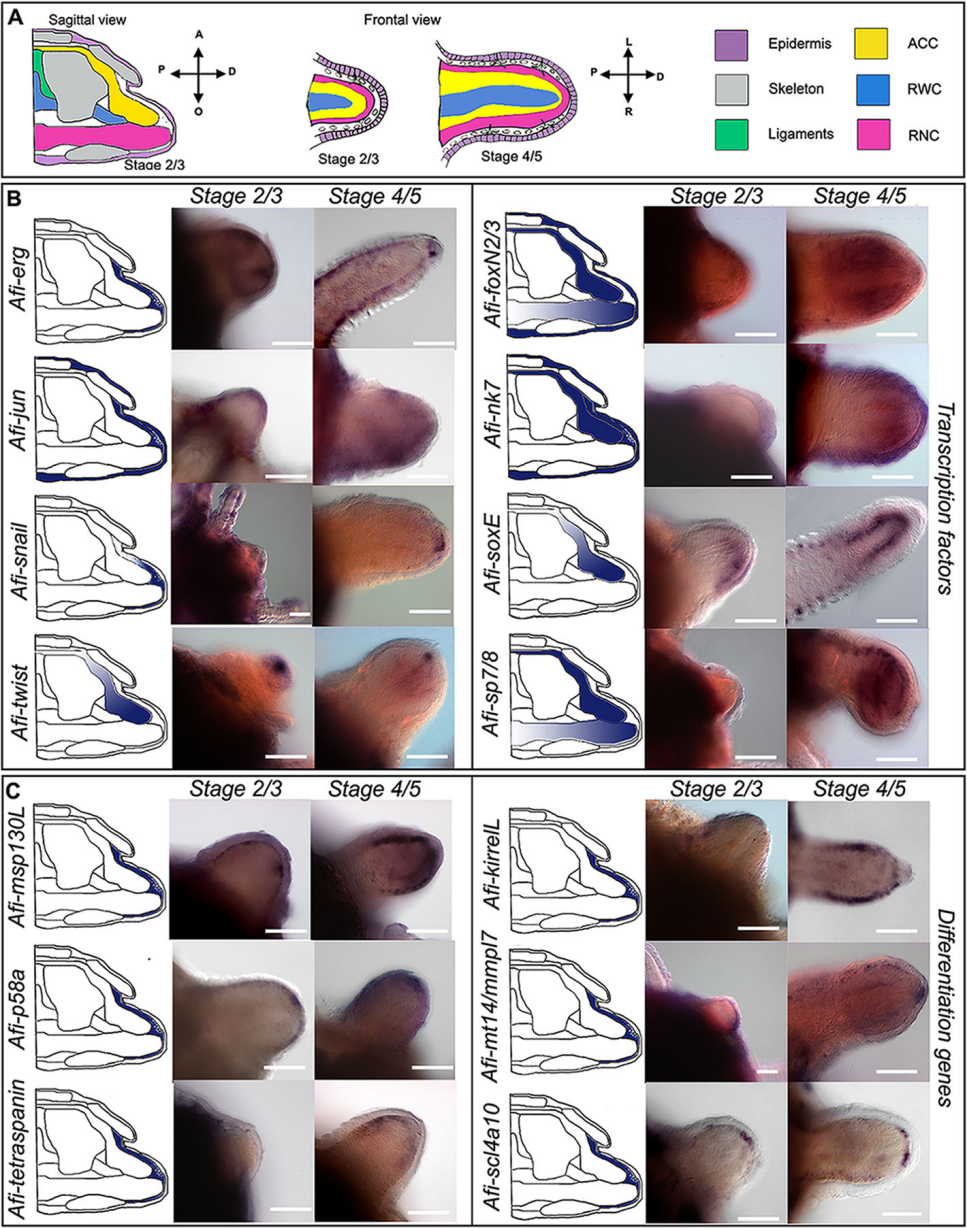
Gene expression at early stages of regeneration (stage 2/3 and stage 4/5). **a** Colour coded sagittal scheme of stage 2/3 and frontal schemes of stages 2/3 and 4/5. Crossed arrows near schematic indicate the axis of the section. Schematics of gene expression in **b** and **c** follow the sagittal scheme as this view allows clear distinction of all tissues. Pictures of gene expression in whole mount are taken in frontal view from the aboral side after orienting the samples, unless otherwise specified. **b** Whole mount ISH at two regenerative stages (stage 2/3 and stage 4/5) as indicated at the top of the columns using antisense probes for transcription factors. Probe name is indicated on the left of the summary schematics. **c** Whole mount ISH at two regenerative stages (stage 2/3 and stage 4/5) as indicated at the top of the columns using antisense probes for known differentiation genes. Probe name is indicated on the left of the summary schematics. In **b** and **c**, summary schematics of expression are based on several images of different focal plane observations of multiple samples; however, only one focal plane is shown here. A, aboral; O, oral; R, right; L, left; P, proximal; D, distal. Dark blue/purple indicates probe-specific signal. Scale bars = 100 μm

*Afi-erg* and *Afi-jun* show staining in the developing dermal layer (Fig. 4) with *Afi-jun* presenting additional staining in the epidermis. *Afi-nk7* stains the epidermis, the developing dermal layer, and the ACC, starting from stage 4/5. *Afi-snail* signal is also in the developing dermal layer, but it is only localized at the distal end of the arm (Fig. 4 and Additional file 2 Fig. S5).

The transcription factors *Afi-soxE* and *Afi-twist* are expressed in the distal ACC epithelium with *Afi-soxE* showing additional staining in the developing lateral arm plates in the proximal region of the regenerates starting from stage 4/5 (Fig. 4 and Additional file 2 Fig. S5).

Both *Afi-foxN2/3* and *Afi-sp7/8* show expression in the ACC epithelium as well as in the tip of the RNC (Fig. 4) with *Afi-foxN2/3* showing additional staining in the epidermis as well (see Additional file 1 Figs. S3 and S5). Lastly, *Afi-sp5* show expression only in the epidermis and *Afi-pax1/9* show no staining at all stages analysed (Additional file 2 Fig. S6). To control for correct functioning of the *Afi-pax1/9* probe, we conducted whole mount in situ hybridization in developing larvae of *A. filiformis*. Here, the probe shows clear expression in a region of the ectoderm of the blastula and in ectodermal cells of the lower arms, adjacent to the mesenchymal cells at the gastrula stage (Additional file 2 Fig. S6).

The other six genes selected are skeletogenic differentiation genes that are either expressed in the skeletogenic cells of developing brittle star and sea urchin embryos [23, 26, 49] or are proteins found in adult skeletal components of sea urchin and brittle star [50–52]. During the early stages of regeneration, *Afi-msp130L*, *Afi-slc4a10*, *Afi-mt14/mmpl7*, and *Afi-p58a* were found expressed specifically in the dermal layer (Fig. 4). *Afi-kirrelL* and *Afi-tetraspanin* only show expression in the dermal layer at stage 4/5, while no signal is detectable before (Fig. 4). Quantitative analysis of six TFs (*Afi-erg*, *Afi-foxN2/3*, *Afi-jun*, *Afi-nk7*, *Afi-snail*, and *Afi-twist*) and of all six differentiation genes agrees with what was observed in ISH (see Additional file 2 Fig. S7 and Table S1). Moreover, most differentiation genes show upregulation of their expression as regeneration progresses (see Additional file 2 Fig. S7 and Table S1).

### Localization of skeletogenic gene expression at late stages of regeneration

**Fig. 5:**
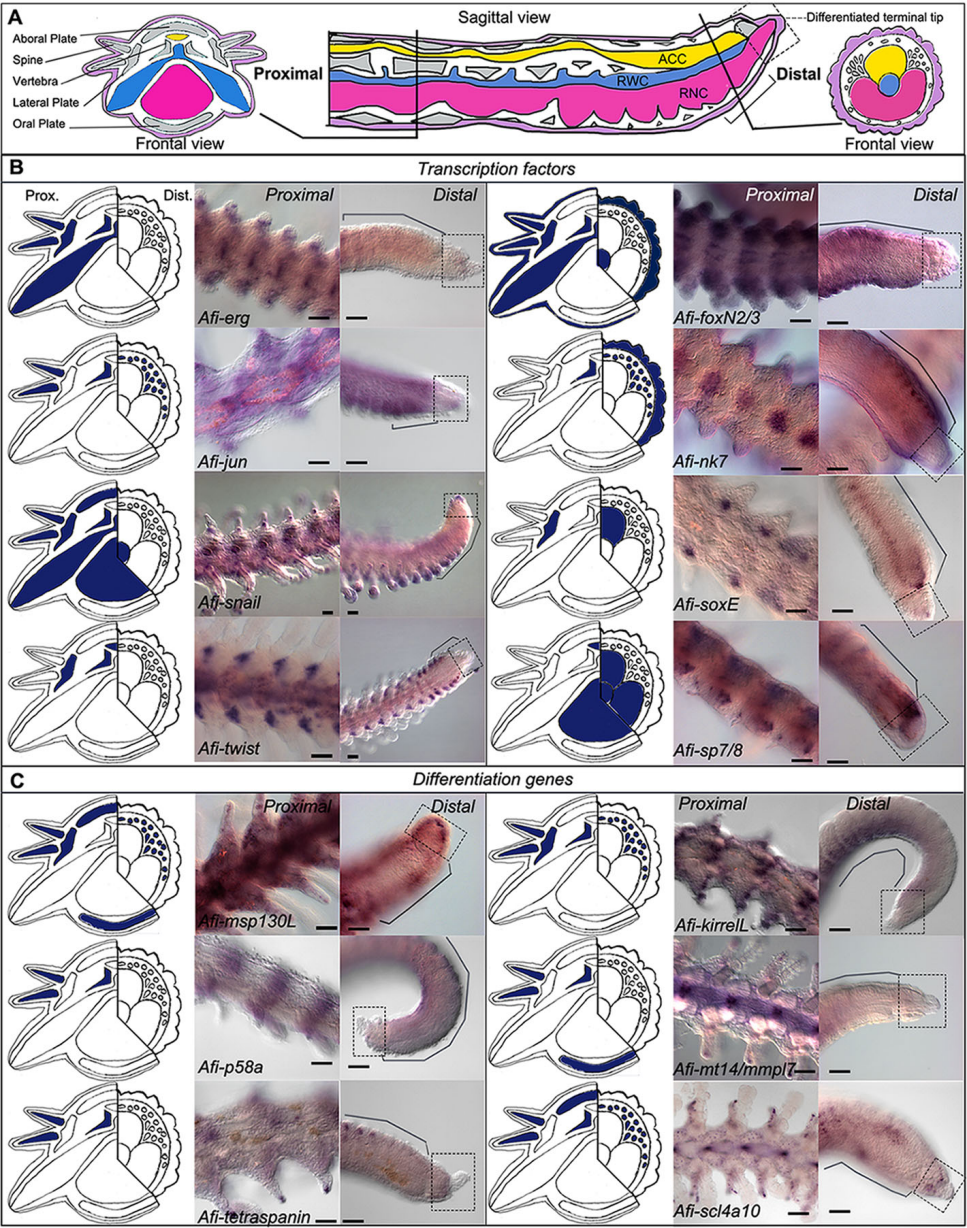
Gene expression at late stages of regeneration. **a** Sagittal schematic of regenerating arm at late stages of regeneration with proximal (Prox.) and distal (Dist.) schematics of cross sections. Purple, epidermis; grey, skeleton; yellow, ACC; blue, RWC; pink, RNC. **b** Whole mount ISH (proximal and distal position as indicated at the top of the columns), using antisense probes for transcription factors. **c** Whole mount ISH (proximal and distal position as indicated at the top of the columns), using probes for known differentiation genes. For **b** and **c**, probe name is indicated at the bottom of the proximal image. On the left of each pair of ISH pictures, there is a summary of expression (blue) in cross schematics for the proximal (Prox.) and distal (Dist.) arm. Summary schematics of expression are based on focal plane observations of multiple samples; however, only one focal plane is shown here. In the distal figures, dotted squares indicate the differentiated terminal structure (terminal ossicle and podium) and black lines indicate the proliferating area. The summary expression data in the schematic is taken from the proliferating area. Scale bars = 50 μm

We then looked at gene expression of the same molecular markers at later stages of regeneration when 50% or more of the arm had already differentiated (DI: differentiation index). At this point, the complex architecture of the skeletal elements starts forming. In general, although many of these genes were expressed ubiquitously in the dermis at early stages, at later stages, the situation is quite different (Fig. 5).

In the distal undifferentiated end of the regenerate proximal to the tip (where the terminal differentiated structures are present), *Afi-jun* is expressed in a broad dermal domain, whereas *Afi-soxE* is again localized to the ACC epithelium. *Afi-nk7* is expressed in both the epidermis and the dermal layer in a repetitive pattern that follows the segments. *Afi-snail*, *Afi-foxN2/3*, and *Afi-sp7/8* show expression in the tip of the RWC, in what is called the terminal podium (see Fig. 5 and Additional file 2 Fig. S6). The latter two also show expression respectively in the epidermis (*Afi-foxN2/3*) and the ACC epithelium and the RNC (*Afi-sp7/8*) (Fig. 5). *Afi-erg*, *Afi-sp5*, and *Afi-twist* are not expressed in this region (Fig. 5 and Additional file 2 Fig. S6).

In proximal segments, the differentiating skeletal elements express various combinations of transcription factors (Fig. 5). For example, *Afi-jun* and *Afi-rreb1* show expression in both vertebrae and spines (Fig. 5 and Additional file 2 Fig. S6). This is also true for *Afi-erg* and *Afi-foxN2/3* which additionally show expression in the lateral arm plates (Fig. 5). *Afi-snail* shows a wider expression in all skeletal elements except the oral arm plates (Fig. 5). *Afi-soxE* and *Afi-twist* show staining, respectively, only in the lateral arm plates, vertebrae, and ACC (Fig. 5). *Afi-nk7* expression is confined to the vertebrae only (Fig. 5). *Afi-sp5*, *Afi-sp7/8*, and *Afi-pax1/9* are not expressed at all in the skeleton (Fig. 5 and Additional file 2 Fig. S6).

Three of the downstream genes analysed (*Afi-msp130L*, *Afi-kirrelL*, and *Afi-scl4a10*) show staining in the dermal layer of the proximal regenerating arm at later stages as well as during early stages. Consistent with their role as terminal differentiation genes, the other downstream genes analysed (*Afi-p58a*, *Afi-tetraspanin*, and *Afi-mt14/mmpl7*) do not show any staining in the distal-most part of the regenerating arm, but only in more developed proximal metameric units.

As for the proximal part, *Afi-msp130L* is the most widely expressed and is present in all external skeletal domains (all arm plates and spines) but not in the vertebrae (Fig. 5). *Afi-p58a* and *Afi-tetraspanin* are restricted to vertebrae and lateral arm plates, whereas *Afi-kirrelL* also shows expression in the spines (see Fig. 5). *Afi-slc4a10* is expressed in vertebrae, spines, and aboral arm plates, and *Afi-mt14/mmpl7* is expressed in spines, vertebrae, and oral arm plates (see Fig. 5).

Altogether, a pattern arises with vertebrae, lateral arm plates, and spines expressing more TFs and downstream genes than oral and aboral arm plates.

Similarly to what shown for early stages of regeneration, quantitative data of six TFs (*Afi-erg*, *Afi-foxN2/3*, *Afi-jun*, *Afi-nk7*, *Afi-snail*, and *Afi-twist*) and of all six differentiation genes at later stages are consistent with the ISH patterns observed (see Additional file 2 Table S1).

### Collagen deposition

The skeletal stroma of the non-amputated arm and the developing dermis of the regenerating arm show large quantities of collagen fibrils and microfibrils (Fig. 1C, D, G; Fig. 2f; Additional file 1 Fig. S2 A, D). Additionally, the above described pocket-like structure of the sclerocyte precursors contains presumptive collagen fibrils, which are comparable in terms of ultrastructural appearance and size with those widespread in the dermal tissue (Fig. 2b). Fibroblasts, cells involved in collagen deposition, have been described in ophiuroids as rather undifferentiated cells that resemble sclerocytes but lack branches and distal processes [17]. As they do not present a clear morphology, we could not distinguish them from sclerocytes or sclerocyte precursors. Therefore, we tried to investigate whether collagen secretion and biomineralization were performed by the same cells, at least during regeneration, as this would allow us to better characterize the role of sclerocytes during regeneration. For this purpose, we selected two collagen genes that previous studies identified as expressed in the dermal layer: *Afi-alpha-collagen* and *Afi-col-L C* [33, 53] and one well-established skeletogenic marker (*Afi-c-lectin*) and performed fluorescent double whole mount in situ hybridizations. Our results on late stages of regeneration show that cells in the skeleton co-express c-lectin and these two types of collagen (Additional file 2 Fig. S8) suggesting that sclerocytes in the regenerating arm are secreting at least these types of collagens.

## Discussion

This work aims to shed light on the origin of sclerocyte precursors and analyse their differentiation process in the regenerating arm of the brittle star *Amphiura filiformis*. Using a combination of ultrastructural and molecular analysis, we show that sclerocyte precursors likely originate from the epithelium of the aboral coelomic cavity. Moreover, we characterize the morphology and molecular signature of these cells as they differentiate. Finally, we show that at advanced regenerative stages unique combinatorial gene expression underlies the patterning of the different skeletal elements. Altogether, our results highlight many similarities between adult and embryonic skeletogenesis in echinoderms and may help to unravel the origin and evolution of the deuterostome skeleton.

### The origin of sclerocytes in the brittle star regenerating skeleton

**Fig. 6:**
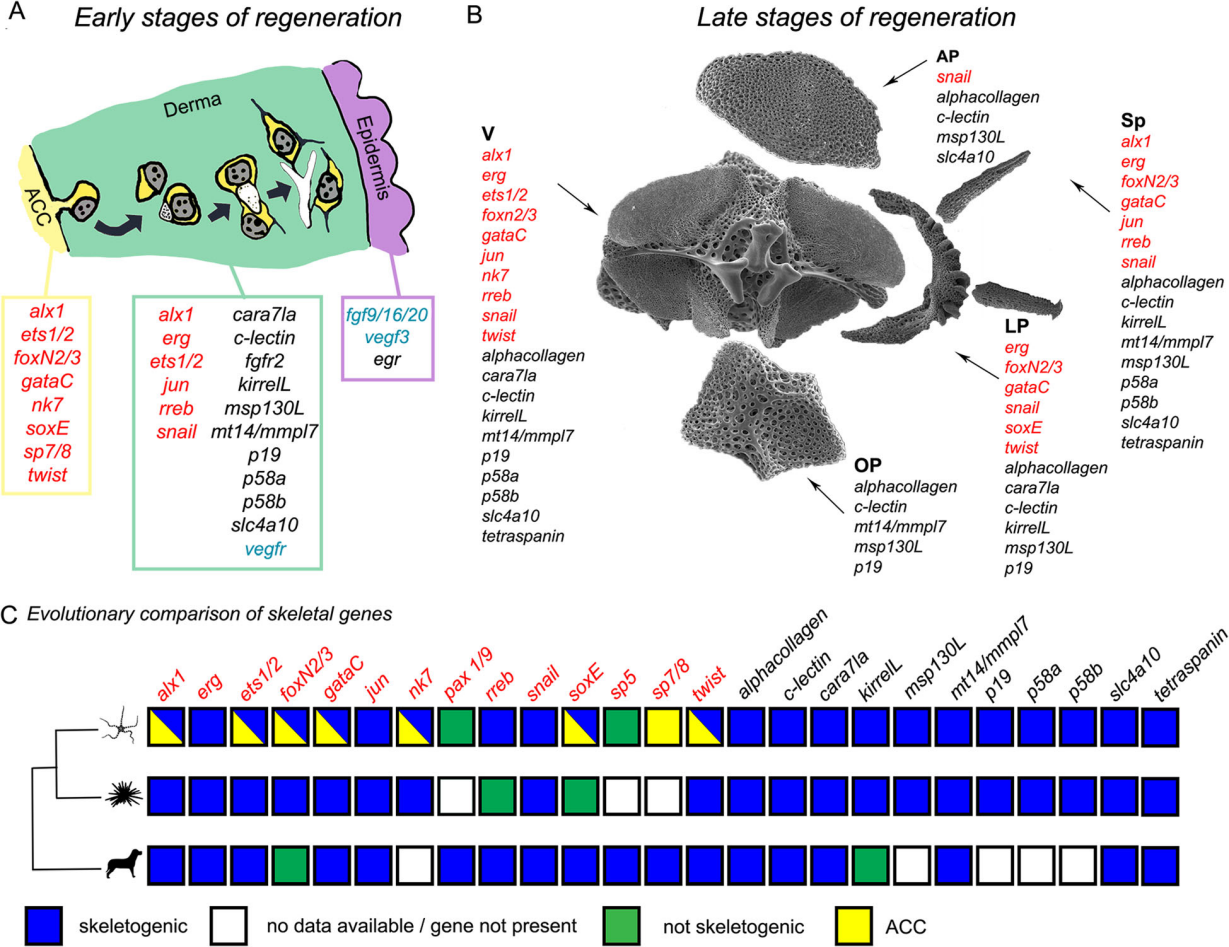
Summary of the origin of skeletogenic cells and the evolution of the molecular signature in deuterostomes. **a** Model of main hypothesis of sclerocyte origin and differentiation at early stages of regeneration (stage 2/3 and 4/5): new sclerocyte precursors detach from the aboral coelomic cavity (ACC) epithelium and differentiate while moving towards the epidermis together with summary of regulatory (red), differentiation (black), and signalling (blue) gene expression of the ACC, the dermis, and the epidermis. Schematics are based on data shown in Fig. 2 and Additional file 1 Fig. S2; gene expression data is based on Figs. 3 and 4 and Additional file 2 Figs. S5 and S6 combined with already published data (References in Table S2). **b** Summary of molecular signature of different skeletal elements at the proximal side of late stages of regenerating arms. Gene expression data is based on proximal differentiating skeletal elements shown in Fig. 5 and Additional file 2 Figs. S5 and S6 together with published data. AP, aboral arm plate; LP, lateral arm plate; OP, oral arm plate; Sp, spine; V, vertebra. **c** Phylogenetic tree with conserved role of skeletogenic genes between the brittle star *Amphiura filiformis* (present work), *Strongylocentrotus purpuratus*, and vertebrates. Genes encoding for transcription factors are in red, and the differentiation genes are in black. For details and relative references, see Additional file 2 Table S2. For this summary figure, we considered the gene present if we could find data on a member of the gene family; therefore, it is not a strict 1:1 orthology, considering also the two rounds of genome duplication occurred at the base of vertebrate evolution

The aim of this work was to understand the origin of sclerocytes as well as their differentiation during arm regeneration in *A. filiformis*, following traumatic amputation. For this reason, we analysed the ultrastructure of and gene expression in cells of the developing dermal layer where the skeleton forms [34]. As cells in the developing dermis had been previously shown to be non-proliferative, part of this work was aimed at finding a potential alternative source. Apart from the developing dermis, there are only four other tissues in the regenerating arm of *A. filiformis*, all of which are proliferative from stage 2/3: the epidermis, the ACC, the RWC, and the RNC [34]. Our gene expression results at early stages of regeneration (stages 2/3 and 4/5; Figs. 3 and 4 and Additional file 2 Fig. S5) combined with previously published data [33] show that the ACC epithelium is characterized by the expression of several TFs, which includes *alx1*, *ets1/2*, *foxN2/3*, *gataC*, *nk7*, *soxE*, and *twist* (Fig. 6a). The expression of several TFs implicated in embryonic skeletogenic lineage specification in the ACC epithelium supports the hypothesis that this tissue could be a potential source of skeletogenic cells among other cell types. Those TFs could be responsible for the specification of cells of the ACC before detachment similarly to the specification of the skeletogenic mesodermal cells of sea urchin and brittle star embryos before ingression. However, the exact regulatory state of specified sclerocyte precursors in the ACC will be revealed by further studies using double in situ and/or single cell sequencing. Once specified, sclerocyte precursors could perform EMT, detaching from the epithelium to enter the developing dermal layer. This hypothesis is supported by our TEM data (Fig. 3b) on serial sections, which show cells likely detaching from the ACC, whereas no cells were captured detaching from the RWC or the RNC in all TEM images analysed. From a mechanical point of view, it would be easier for cells to detach from the ACC epithelium due to the lack of a thick basal lamina in the regenerating tip, which instead is present in the regenerating RNC and RWC. The RWC, particularly, is normally provided with a very thick basal lamina [54] (in Additional file 1 compare Fig. S4 D with B, F and G’). Moreover, cells in the oral epithelium of the ACC appear more loosely connected (Additional file 1 Fig. S2 and S4 A and A’). The ACC has already been proposed as a possible source of cells during regeneration of both *A. filiformis* and *Ophioderma longicaudum* [36] and of other echinoderms as well [11, 55, 56]. In fact, during crinoid and starfish arm regeneration, as well as in holothuroid visceral regeneration, cells detach from the epithelia of the regenerating coelomic (somatocoel) compartment and ingress the underlying mesenchymal tissue. Nevertheless, the RWC has also been previously proposed as a source of cells during brittle star regeneration [57].

To corroborate our hypothesis, which is based on static TEM and ISH images, it will be fundamental to perform lineage tracing experiments and develop tools for knockouts of TFs expressed in these cells to confirm their role in sclerocyte specification.

### Progression of sclerocyte differentiation during regeneration

Assuming that the cells described detach from the epithelium of the ACC, our work suggests that, as they migrate towards the epidermis, they start differentiating as in the model presented in Fig. 6a and in Fig. 2. In fact, mesenchymal cells closer to the axial structures (and the ACC) look less differentiated than those adjacent to the epidermis, although they display some characteristics of mature sclerocytes of other ophiuroids and of cidaroid echinoids: patchy nucleus, abundant RER, several mitochondria, and a secondary boundary layer [17, 18, 32, 58]. We hypothesize that most of these cells are sclerocyte precursors.

Consistent with this hypothesis, we show that cells close to the axial structures already express skeletogenic genes, such as *c-lectin*, *alx1*, and *msp130L* (Fig. 3).

As these cells move towards the epidermis, they increase the amount of cytoplasm, project cytoplasmic branches, and develop a pocket-like structure that delimits an extracellular space, which is initially filled with presumptive collagen fibrils and then becomes more electron-transparent (in Fig. 2 compare b with c). As TEM images show, the cytoplasmic projections of the pockets later fuse with those of other nearby cells and this is where spicules begin to form, similarly to what happens in both sea urchin and brittle star embryos (Fig. 6a and Fig. 2) [59, 60]. Consistent with the acquisition of cell differentiation features, cells in the dermal layer express a large set of skeletogenic differentiation genes (for summary see Fig. 6a) and have a specific regulatory state (combination of TFs) that partially overlaps with that of the ACC. The two transcription factors *alx1* and *ets1/2* in common between the ACC and the differentiated sclerocyte are known in the sea urchin embryo to constitute the key regulators of the skeletogenic gene battery and cellular features [61]. Therefore, our data suggest that the programme underlying the cellular mechanism of spicule formation within a cellular syncytium takes place not only in embryonic but also in post-metamorphic skeleton development, and it is a shared feature with other echinoderm classes (e.g. Echinoidea). Consistent with this, we also show that *Afi-kirrelL*, which in sea urchins is responsible for the fusion of filopodia necessary for larval skeleton deposition [62], is expressed from stage 4/5 in the dermal cells, when skeletal primordia are actively deposited [34]. Differently from sea urchin embryos, in adult regenerating arms of *A. filiformis*, sclerocyte precursors do not produce long and thin filopodia, but only very short extensions. This is possibly linked to the physical constrictions experienced by the population of skeletogenic cells, which in the embryo are freely moving in a cell-deprived fluid of the blastocoel, but in the regenerating arm are highly crowded in an extracellular matrix-rich dermal layer.

Finally, cells reach the region just beneath the epidermis where they appear scattered in a dermal layer filled with collagen fibrils and growing spicules. At this stage, cells acquire a more differentiated morphology, resembling that of mature sclerocytes: the cytoplasm surrounding the nucleus is reduced, with cell projections extending towards the growing spicules (Fig. 2e, f). The expression of several biomineralization genes specifically in the dermal layer is consistent with the fact that in this location cells are indeed producing the skeleton (Figs. 6a, 3, and 4) [33].

Recent studies have shown that *fgf9/16/20* and *vegf3* signals from the epidermis of *A. filiformis* are necessary for the formation of biomineralized spicules during regeneration [25]. This signal could drive the migration and final differentiation of sclerocyte precursors and initiation of deposition of the biomineralized spicule that we show ultrastructurally (see Fig. 2e, f and Additional file 1 Fig. S2 D).

It is worth noting that the process of apparent migration towards the epidermis and subsequent differentiation just described is valid for the external skeletal elements of the arm (arm plates and spines). The sclerocyte precursors of the vertebrae, which have been briefly described here as the cells present in between the ACC and the RWC at stage 4/5 (Additional file 1 Fig. S2 C), might undergo slightly different cellular and molecular processes, as they never come in contact with the epidermis. Here, other tissues, such as the RWC, might function as “inductive elements” promoting sclerocyte differentiation.

Besides sclerocytes, very few cell types were observed in the derma. Presumptive pigment cells, granulated cells, and nerve cells identified in the mature stroma of the skeleton were not found at early stages of regeneration, suggesting that they might populate this tissue only later. Fibroblasts, which are normally involved in collagen secretion, could not be unambiguously identified either in the mature or in the regenerating arm although large quantities of collagen and microfibrils fill the stroma and the developing dermis. Since in the literature fibroblasts are described as morphologically similar to sclerocytes, we wanted to further investigate if in *A. filiformis* they are indeed two distinct cell types [17]. Our results at advanced stages of regeneration show that cells in the skeleton co-express two collagen genes with the skeletogenic marker *c-lectin* (Additional file 2 Fig. S8). This suggests that sclerocytes also play a role in collagen secretion and gives a molecular significance to the ultrastructural observations made by Märkel and Röser [17] and Heatfield and Travis [32]. Moreover, two other collagen genes (*Afi-col-L B* and *C*) have recently been shown to be expressed in the developing dermal layer of *A. filiformis* at early regeneration stages (3/4) and in the skeleton at later stages [53].

Altogether, our cellular and molecular findings suggest a conserved mechanism of sclerocyte differentiation during development and regeneration in echinoderms. This comprehensive, molecular, and ultrastructural study forms the basis for future functional work.

### Unique combinatorial gene expression underlies the patterning of the different skeletal elements during regeneration

Once sclerocytes are fully developed, they start secreting calcium carbonate in the form of single spicules. These spicules then grow and fuse to form the 3D meshwork typical of the stereom and the final ossicles of the arm. If we look at gene expression at this advanced stage of regeneration, we notice two very interesting patterns arising.

First, we observe that in the distal-most part of the regenerating arm, right underneath (proximal to) the differentiated terminal ossicle and podium, gene expression of seven genes highly resembles that of early stages (*Afi-jun*, *Afi-nk7*, *Afi-soxE*, *Afi-sp7/8*, *Afi-msp130L*, *Afi-kirrelL*, and *Afi-scl4a10*). This further supports the idea that echinoderm arm regeneration follows the distalization-intercalation mode of regeneration as proposed previously [34, 63].

Secondly, although many of the genes analysed showed a broad dermal domain at early stages of regeneration, at later stages in the proximal region (older and most developed segments), we observed that different sets of genes are expressed in different skeletal elements. In particular, vertebrae, lateral arm plates, and spines show expression of many more genes (both TFs and differentiation genes) than oral and aboral arm plates (Figs. 5 and 6b). This is consistent with the degrees of stereom organization complexity and densities in these different skeletal elements (Additional file 2 Fig. S9). Vertebrae, for example, show the highest degree of complexity, with different pore sizes and rod thickness in the area where muscles or joints are present as well as grooves for the passage of the RNC, the RWC, and the ACC. Lateral arm plates, which are shaped like a half-moon, also possess highly dense protrusions where spines attach. Spines themselves have a conical calcite structure and display an array of different shapes (long and thin, slightly thicker, and with hammer-shape tip) (see Fig. 6b). By contrast, the oral and aboral arm plates have a simpler, almost flat structure with even pore size and rod thickness across the plate. Additionally, besides sclerocytes, the set of cell types present in different mature skeletal elements can be different ([54] and personal observations). In conclusion, we suggest that the differences in molecular signatures may reflect the complexity of morphologies of the different skeletal elements as well as anatomical position.

Moreover, our optimized technique for WMISH sectioning in resin/wax could be a powerful tool to verify whether there is a correlation between different stereom densities and molecular signatures. This might be of particular interest in the field of palaeontology since the pore size and thickness of trabeculae vary depending on the type of soft tissues attached to the skeleton, and hence on their specific functions [64]. It would be also interesting to explore the role of signalling coming from different tissues in skeleton development. It has been recently confirmed that signalling from the epidermis is crucial for the onset of biomineralization in arm plates. However, we know very little about signalling from other tissues [25]. The vertebrae, for example, which have no contact with the epidermis, could receive signalling from other tissues, such as the ACC, the RWC, and the RNC, and this could orchestrate the formation of the different grooves.

### Evolutionary origin of skeletogenesis in deuterostomes

We have so far discussed in detail skeletal regeneration in the mature arms of *A. filiformis* and highlighted some similarities with sea urchin development. Other deuterostome groups, such as vertebrates, are in some cases able to regenerate the endoskeleton of their appendages [65–67].

We therefore analysed a number of genes that are known to have a role in both sea urchin skeletal development and in vertebrate chondrogenesis and/or bone formation. We compare skeleton formation in echinoderms with both cartilage and bone formation in vertebrates as the earliest vertebrate skeleton was likely made of unmineralized cartilage [68].

There are clear differences in the way that vertebrates and echinoderms produce their biomineralized tissues; notably, they use different minerals: calcium phosphate for vertebrates and calcium carbonate for echinoderms [68]; the vertebrate crystallization pathway involves mineralization of extracellular matrix, while echinoderm mineralization is enclosed in a cell syncytium [69]. These differences are reflected in the evolution of clade-specific mineralization genes, such as the echinoderm skeletogenic matrix (SM) genes, or the specific co-option of the vascularization programme regulated by VEGF in sea urchins [70]. Mineralization in both vertebrates and echinoderms, however, takes place in an extracellular space, and it is secreted by mesenchymal cells. Our aim is not to compare individual cell types across phyla (i.e. echinoderm sclerocytes with vertebrate osteoblasts and chondrocytes), which might be ineffective given the long independent evolutionary history of these two deuterostome clades (at least 540 millions of years), but rather to explore the extent of conservation of the basic deuterostome biomineralization toolkit present in the last common ancestor before the divergence of echinoderms and chordates.

Figure 6c summarizes the occurrence of our set of genes together with other published data in vertebrates, sea urchin, and *A. filiformis* and their roles in skeleton formation (mineralized or not).

Twenty-three out of 25 of these genes are expressed in the skeletogenic tissues of *A. filiformis* during regeneration. Of those 23 genes, 20 also show expression in the skeletogenic mesoderm of *S. purpuratus* embryos, indicating a conserved role in both development and regeneration of the skeleton (for references see Additional file 2 Table S2). Consistent with the studies carried out by Czarkwiani and colleagues [25], this observation supports the idea that skeletogenesis during regeneration highly resembles embryonic skeleton formation not only from a cellular but also from a molecular point of view.

Fifteen of the 25 genes chosen (*alx1*, *erg*, *ets1/2*, *gataC*, *jun*, *rreb*, *snail*, *soxE*, *twist*, *alpha-collagen*, *cara7la*, *c-lectin*, *mt1/4/mmpl7*, *slc4a10*, and *tetraspanin*) also play a role in skeleton (bone or cartilage) formation in vertebrates (for references see Additional file 2 Table S2). Additionally, we specifically considered three vertebrate sclerotome/chondrogenic markers, i.e. *pax1*, *sox8/9/10*, and *sp7* or *osterix* [43–48, 69, 71]. *Pax 1/9*, the echinoderm homologue of vertebrate *pax1*, showed no staining [43, 47]. *Sp5* and *sp7/8* are the sea urchin homologues of vertebrate *sp7* (or *osterix*) [44, 46, 71]. *Afi-sp5* was expressed only in the epidermis (at early stages of regeneration); however, *Afi-sp7/8* was expressed at all stages of regeneration in the ACC and in the RNC. Finally, *soxE*, the sea urchin orthologue of vertebrate *sox8*, *sox9*, and *sox10* genes, was localized in the ACC at early stages and in the lateral arm plates at later stages of regeneration. The expression of *Afi-sp7/8* and *Afi-soxE* in the ACC agrees with our hypothesis on the coelomic origin of sclerocytes; however, *Afi-soxE* skeletogenic role is additionally confirmed by its expression in skeletal elements at later stages of regeneration.

These observations, together with the expression of *alx1* and involvement of *fgf* signalling [25], identify an overlap in the molecular pathways used by sclerocytes in *A. filiformis* and skeleton-forming cells in vertebrates. As vertebrate osteoblasts and chondrocytes and echinoderm sclerocytes all derive from mesenchymal precursors, we cannot rule out that this overlap could be due to a common mesenchymal regulatory programme rather than a conserved role in skeletogenesis. The use of regulatory modules encoding for specific cell/tissue processes, such as EMT or tubulogenesis, is in line with what recently presented for the regulatory programme of vertebrate endothelial cells and echinoderm sclerocytes, which share several genes [70]. Our cellular and molecular characterization of adult echinoderm sclerocytes will help further investigating the origin and evolution of the deuterostome skeleton.

## Conclusions

This study provides cellular and molecular insights into the possible origin and differentiation of sclerocytes in brittle star arm regeneration following traumatic amputation. Our findings strengthen the hypothesis that adult regeneration in echinoderms re-uses a developmental programme. Moreover, our molecular data identify several commonalities between echinoderm skeletogenesis and skeletal development in other deuterostomes.

## Materials and methods

### Animal maintenance and handling

Adult (disc diameter ~ 0.5 cm) specimens of *Amphiura filiformis* O. F. Müller, 1776 were collected at the Sven Lovén Centre for Marine Sciences in Kristineberg (Sweden). Experimental animals were kept in aerated aquaria of artificial seawater (ASW) (Instant Ocean®) at 14 °C and 34‰ salinity, and chemical-physical ASW parameters were constantly checked. Animals were fed twice a week with Microvore Microdiet (Brightwell Aquatics). During all manipulations, animals were anesthetized in solution of 3.5% MgCl_2_ in distilled water (dH_2_O) and ASW (1:1). To minimize stress, a maximum of two arms out of five per brittle star were artificially amputated with a scalpel at the level of natural autotomy planes (i.e. between plates/intervertebral articulations). Despite our efforts to mimic pseudo-autotomic conditions, in the present work, we specifically investigated post-traumatic regeneration, which might partially differ from natural post-autotomic regeneration [72, 73]. After amputation, the animals were returned to the aquarium and left to regenerate for a given amount of time to reach the desired stages: 2, 3, 4, 5, 50% differentiation index (DI) and 95% DI [14, 34]. Following this step, regenerating arms (including few segments of stumps) were collected for whole mount in situ hybridization (WMISH) experiments and microscopy analyses.

### Transmission electron microscopy

Samples for transmission electron microscopy (TEM) were processed as described by Ferrario et al. [54]. For each stage (non-regenerating, stage 2, stage 3, stage 4, stage 5, 50% DI, 95% DI), at least four samples were used for semi-thin sectioning; the most informative were then used for ultrathin sectioning (at least two per stage). For each sample, we cut approximately 40 sections, a subsample of which were observed at the TEM. Semi-thin and ultrathin sectioning was performed with a Reichert Jung Ultracut E. Serial semi-thin sections (~ 1 μm thick) were cut with glass knife. The most informative sections were stained with crystal violet and basic fuchsin and observed under a Jenaval light microscope provided with a DeltaPix Invenio 3S 3M Pixel CMOS Camera and DeltaPix Viewer LE Software. Ultrathin sections (~ 70–80 nm) were cut with glass knife, mounted on copper grids (300–400 mesh), and stained with 1% uranyl acetate and lead citrate in order to be observed and photographed in either a Jeol 100SX or a Zeiss LEO 912AB transmission electron microscope.

### Scanning electron microscopy

Single ossicles were isolated from regenerating and non-regenerating arm samples using 1 M NaOH until soft tissues were completely dissolved, and they were then washed in dH_2_O several times. The ossicles were first photographed using a light microscope (Leica MZ75, with Leica CLS 150XE lights, provided with a camera Leica Digilux 18.102) then mounted on stubs, gold-coated (Sputter Coater Nanotech), and observed with a LEO-1430 scanning electron microscope (SEM).

To better visualize advanced skeleton formation, one of the late stages regenerating sample was fixed in Bouin (saturated aqueous solution of picric acid, 37% formaldehyde, and glacial acetic acid), embedded in wax following standard procedures [63], and then longitudinally sectioned to reach the sagittal plane. Wax was then removed with several washes in xylene at room temperature (RT), and the sample was prepared for SEM analysis as follows: washes in 100% ethanol (EtOH); washes in 3:1, 1:1, 1:3 100% EtOH and hexamethyldisilazane (HMDS); and finally 100% HMDS. The sample was left to air-dry, mounted on a stub, gold-coated (Sputter Coater Nanotech), and observed with a LEO-1430 SEM.

### Candidate gene identification

Genes of interest were selected from the literature based on their involvement in the skeletogenic process of echinoderm species or vertebrates. Both transcription factors and differentiation genes were considered. Specific sequences were retrieved from Echinobase (http://www.echinobase.org/Echinobase/) or NCBI. In order to obtain the corresponding gene sequences in *A. filiformis*, BLAST-X was performed using the *A. filiformis* transcriptome [24]. The top-ranking blast sequences were then blasted back on Echinobase or NCBI to ensure a reciprocal relationship and distinguish between genes belonging to the same orthologous group and analogous genes. Specific primers were designed using PRIMER3 Software version 0.4.0 (http://primer3.ut.ee/), optimizing the following parameters: max 3′ stability was set at 8.0 and max polyX at 3. Their specificity was checked by performing a BLAST-N to the *A. filiformis* embryo transcriptome [24].

### Cloning and probe synthesis

All genes used for spatial expression analysis by WMISH were PCR amplified from *A. filiformis* cDNA pool and cloned in the pGEM-T easy vector system (Promega) or Topo PCR cloning system (Invitrogen) according to the manufacturer’s instructions. Identity of the fragment was confirmed by sequencing. Antisense probes labelled with DIG (Roche) or DNP were synthesized as previously described [23]. Primers and clone length are listed in the supplementary Additional file 2 Table S3.

### Whole mount in situ hybridization

WMISH allowed the visualization of the expression of genes of interest. Regenerating arms (including a few segments of stumps) for WMISH were fixed in 4% paraformaldehyde (PFA) in 1× MABT (0.1 M Maleic Acid pH 7.5, 0.15 M NaCl, 0.1% Tween-20) buffer overnight at 4 °C and washed several times with 1× MABT. For long-term storage, the regenerates were left in 100% methanol at − 20 °C. Arms were first rehydrated with graded ethanol washes (70%, 50%, and 30%), then washed three times in 1× MABT buffer and pre-hybridized in hybridization buffer (HB: 50% deionized formamide, 10% PEG, 0.05 M NaCl, 0.1% Tween-20, 0.005 M EDTA, 0.02 M Tris pH 7.5, 0.1 mg/mL yeast tRNA, 1× Denhardt’s solution, DEPC-treated water) for 1 h at 50 °C.

Next, the arms were put in HB containing 0.04 ng/mL of the antisense probe for 3–7 days at 50 °C (for fluorescent in situs (FISH), two probes, one DIG labelled and one DNP labelled, were added). The arms were post-hybridized in fresh HB without probe for 1 h at 50 °C, then washed once in 1× MABT at 50 °C and once at RT. The samples were then washed three times in 0.1× MABT and once in 1× MABT at RT. Samples were placed in blocking buffer (1× MABT, 0.5% goat serum) for chromogenic in situ (ISH) or in PEBR (0.5% Perkin Elmer Blocking Reagent in 1× MABT) for FISH for 30 min. The arms were then incubated in 1:1000 anti-DIG AP antibody solution in blocking buffer overnight at 4 °C for ISH or in 1:1000 anti-DIG peroxidase (POD) antibody solution in PERB for FISH. Next, they were washed five times in 1× MABT. Samples for ISH were then washed two times in alkaline phosphatase buffer (0.1 M Tris pH 9.5, 0.1 M NaCl, 0.05 M MgCl_2_, 1% Tween-20, 1 mM Levamisole in dH_2_O); then, the staining solution was added (AP buffer, 10% DMF, 2% NBT/BCIP (Roche ready mix)) for chromogenic detection. Staining was monitored under the dissecting scope and stopped at the desired time using 1× MABT with 0.05 M EDTA followed by three washes in 1× MABT. The samples were then placed in 50% glycerol for long-term storage at 4 °C.

Samples for FISH were, instead, washed in amplification diluent for 15 min at RT and stained for 15 min in 1:400 cyanine 3 (Cy3) in amplification diluent. To remove the staining solution, arms were then washed in 1% hydrogen peroxide in 1× MABT for 30 min at RT, and five times in 1× MABT buffer at RT. They were then left in PEBR for 30 min and incubated in 1:1000 dilution of anti-DNP-POD in PEBR overnight at 4 °C. For the second staining, arms were washed in amplification diluent for 15 min at RT and then stained for 15 min in 1:400 cyanine (Cy5) in amplification diluent. Finally, arms were washed five times in 1× MABT at RT and then left in 1× MABT for imaging.

### Imaging of WMISH and sectioning

For whole mount observation, the samples were kept in 50% glycerol and then mounted onto glass slides oriented on the oral and/or aboral side. For differential interference contrast (DIC) images, the Zeiss AxioImager M1 microscope was used together with a Zeiss AxioCam HRc camera. Images were processed using Adobe Photoshop and Fiji. After whole mount imaging, some samples were sectioned for better resolution of the signals following two protocols. The first set of samples were washed twice in 1× MABT, decalcified overnight in 0.5 M EDTA (pH 8.0) at 4 °C; then, they were post-fixed in 4% PFA in 1× MABT for 30 min. A second set of samples were decalcified in 1:1 (v/v) solution of 0.6 M NaCl and 4% l-ascorbic acid (both dissolved in dH_2_O) and post-fixed in 2% glutaraldehyde in 1× MABT.

All samples were then washed twice in 1× MABT, dehydrated with an increasing series of ethanol washes (30%, 50%, 70%, 95%, 100%) of 15 min each followed by two 15-min washes in Histoclear (Histoclear, Biosciences Stores) at RT and one at 60 °C. The samples were then washed three times in liquid wax (Shandon Histoplast paraffin wax) for 30 min and left to solidify overnight. They were cut in sections of 8 μm thickness with a Leica RM2155 microtome and placed on glass slides. The latter were prepared for imaging with two washes of 15 min in Histoclear (Sigma) and mounted with Histomount (Sigma). Both treatments produced similar results.

For confocal images of fluorescently labelled samples, the Leica TCS SP2 confocal laser scanning microscope was used and the LAS-AF software implemented to capture the image stacks. For confocal imaging, 90 z-stacks at 1 μm thickness were collected. Images were processed using Fiji and Adobe Photoshop.

### Nanostring nCounter

Nanostring nCounter analysis system (Nanostring Technologies, Seattle, WA, USA) (Geiss et al., [95]) was used for quantitative gene expression of most of the skeletogenic genes analysed (*Afi-alx1*, *Afi-erg*, *Afi-ets1/2*, *Afi-foxN2/3*, *Afi-gataC*, *Afi-jun*, *Afi-nk7*, *Afirreb1*, *Afi-snail*, *Afi-tbr*, *Afi-twist*, *Afi-alpha-collagen*, *Afi-c-lectin*, *Afi-cara7La*, *Afi-kirrelL*, *Afi-msp130L*, *Afi-mp14*, *Afi-p19*, *Afi-p58a*, *Afi-p58b*, *Afi-slc4a10*, *Afi-tetraspanin*) as described in Czarkwiani et al. [25]. For each sample, 100 ng of total RNA was used, which was extracted from 10 regenerating (without stump) and non-regenerating arms using the RNeasy Micro Kit (Qiagen). The experiments and analysis were carried out according to manufacturer’s instructions. Additionally, the results of the quantification were normalized using the chosen internal standard genes (normalization factor obtained from geometric mean analysis of each lane; chosen internal standard genes: *Afi-Cytchrmeb*, *Afi-Ncbp1*, *Afi-Tfb1m*, *Afi-Ubc*, and *Afi-Ubq* [27];). For relative expression analysis, each gene normalized value was divided by the maximum normalized value for that gene and multiplied by 100.

## Supplementary Information

**Additional file 1: Figure S1.** Ultrastructural analysis of dermal cells at stage 4/5 of regeneration. A) TEM micrographs of a morula-like (or spherule) cell under the epidermis characterized by the presence of large inclusions, indicated by arrowhead. B) TEM micrograph of a phagocyte showing large nucleus with evident nucleolus, phagosome, indicated by arrowhead, and long cytoplasmic projections. Both cells have morphologies that resemble adult coelomocytes. Scale bars = 2 μm. EP = epidermis; RNC = radial nerve cord. **Figure S2.** Mesenchymal cells adjacent to the epidermis appear more differentiated proximally (closer to the amputation plane) then distally (at the tip) as well as in later stages of development (stages 4/5) compared to early ones (stages 2/3). Top left, frontal section schematics of a regenerating arm at stage 2/3 or 4/5. Top right, cross sections of stage 2/3 and 4/5 where letters in red squares indicate the position of TEM micrographs at the bottom. The area highlighted in blue/green is the dermal layer. A) Mesenchymal cells at stage 2/3 at the distal-most tip of the regenerate. Red arrow shows the presence of fibrils. B) Mesenchymal cells at stage 4/5 in the area right next to where ACC and RNC meet on either side of the RWC. C) Mesenchymal cells at stage 4/5 in the distal-most area of the regenerate in-between the ACC and the RWC. Red triangles indicate phagocytes, red circle indicates apoptotic cell, with characteristic nucleus. D) Mesenchymal cell at stage 4/5 in the area right next the epidermis (EP). Asterisk indicates cytoplasmic pocket. ACC = aboral coelomic cavity, RNC = radial nerve cord, RWC = radial water canal, St = stage, scale bars = 2 μm. **Figure S3.** Vertebral primordia at late stages of regeneration form between the ACC and the RWC. A) Semi-thin cross section of a late stage regenerating arm. B) Schematics of A. Purple= epidermis, Pink= RNC, Blue=RWC, Yellow= ACC, Grey= skeletal tissues. **Figure S4.** Ultrastructural analysis of different tissues of regenerating arms shows that the ACC is lacking a basal lamina and is less compact then other tissues of the regenerating arm. A) Semi-thin cross section of the tip of a regenerating arm. Coloured lines indicate the borders of the different structures (purple = epidermis, yellow = ACC, blue = RWC, pink = RNC). Black arrows indicate where cells of the ACC appear loosely connected. Yellow line is dotted where ACC borders are hard to distinguish. Black dotted square indicates approximate location of A’. Asterisk marks the ACC lumen. A’) TEM micrograph of the ACC, red arrows indicate where cells of the ACC appear loosely connected. Yellow line is dotted where ACC borders are hard to distinguish. Red dotted square indicates approximate location of A”. B) aboral coelomic cavity (ACC); C and D) radial water canal (RWC); E and F) radial nerve cord (RNC); G epidermis (EPI); G’) is a magnification of G as indicated by the dotted red square. Arrowheads indicate basal lamina, when present. Scale bars of A and B are 10 μm, other scale bars are 2 μm.

**Additional file 2 **: **Figure S5.** Details of spatial gene expression at early stages of regeneration show that many skeletogenic TFs are expressed in the ACC. Top of the figure shows schematics of sagittal, frontal and sections of early regenerating arm. First column shows schematic summary of gene expression as shown in Fig 4. For *Afi-foxN2/3, Afi-twist* and *Afi-snail* sections of WMISH are shown in sagittal and/or cross session. For *Afi-soxE* different focal planes in frontal view from aboral side are shown, while *Afi-sp7/8* is imaged in semi-frontal view with focus on the regenerate tip. The arms are oriented aborally or orally for imaging and different focal planes inform on whether the tissue shown is the aboral epidermis, the ACC or RWC when imaged from the aboral side; or the oral epidermis, the RNC or the RWC when imaged from the oral side. All front views depicted here are imaged from the aboral side unless otherwise specified. Crossed arrows indicate the orientation of the section: A= aboral, O= oral, R= right, L= left, P= proximal, D= distal. **Figure S6.** Spatial gene expression at early and late stages of *Afi-cara7la*, *Afi-rreb1*, *Afi-sp5*, *Afi-pax1/9 and Afi-foxN2/3*. Columns on the left show the schematic of the arm in sagittal view for early stages; and in cross view for proximal and distal regenerates at late stages. Blue colour indicates the detected gene expression. Low-right images show the result of whole mount ISH of *Afi-pax1/9* at two embryonic developmental stages, where the colorimetric staining is present in ectodermal cells. St. = stage. **Figure S7.** Levels of expression of genes in regenerating and non-regenerating arms. Graph shows the relative expression of skeletal genes in non-regenerating arms and at different stages of regeneration. Abundance of transcripts has been evaluated in 100 ng of total RNA using nCounter (Nanostring) technology. Relative expression (%) has been calculated using normalized counts per 100 ng of RNA relative to the maximum of expression for each gene. Non-regenerating arms (NR), 24 hours post amputation (24hpa), Stage 3 (St3), Stage 4 (St4) and Stage 5 (St5). **Figure S8.** Fluorescent in situ hybridization showing co-localization of *Afi-c-lectin* with collagen genes in the skeletal elements. FISH of *Afi-c-lectin* (green) with collagen genes *Afi-alpha-collagen* (red - right) and *Afi-col-L C* (red - left) in regenerating arm at late stages. Large images are confocal maximum projections. Small images are enlargements of a single Z-stack slide and single fluorescent channel, as specified, with DAPI (blue) showing the coexpression of the two genes. **Figure S9.** Different skeletal structures. SEM analysis in *A. filiformis* mature skeletal elements reveals different stereom structures and densities. A) Stereom of the aboral arm plate embedded in connective tissue shows large pores. B) Stereom of the vertebra from the point where the muscle attaches to it shows small pores. C) Compact stereom of the vertebral condylus. D) Median point where the left and the right vertebral halves fuse together during skeletal development. Scale bars: A = 20 μm; B = 30 μm; C and D = 10 μm. **Table S1.** Levels of gene expression during regeneration. Nanostring normalized counts for skeletogenic genes in non-regenerating arm (NR), 24 hours post amputation (24hpa), 48 hours post amputation (48hpa), 72 hours post amputation (72hpa), Stage 3 (St3), Stage 4 (St4), Stage 5 (St5), proximal part of a 50% regenerated arm (50% prox) and distal part of a 50% regenerated arm (50% dist). **Table S2.** Summary table and references of comparison of gene expression in the sea urchin *S. purpuratus* (Spu), in the brittle star *A. filiformis* (Afi) and in vertebrates (Vert) [23–26, 28, 31, 33, 34, 40–49, 51, 74–94]. The data are used in the summary Figure 6. **Table S3.** List of primers used to amplify and clone specific fragments of *A. filiformis* genes and to produce antisense probes for WMISH. F – forward primer, R – reverse primer, O – outer, I – inner, bp – base pair.

## Data Availability

All data generated or analysed during this study are included in this published article and its supplementary information files.

## Abbreviations

ACC: Aboral coelomic cavity
ASW: Artificial sea water
dH_2_O: Distilled water
DI: Differentiation index
DIC: Differential interference contrast
EdU: 5-Ethynyl-2′-deoxyuridine
EMT: Epithelial-to-mesenchymal transition
FISH: Fluorescent in situ hybridization
GRNs: Gene regulatory networks
HB: Hybridization buffer
HMDS: Hexamethyldisilazane
ISH: In situ hybridization
RER: Rough endoplasmic reticulum
RNC: Radial nerve cord
RT: Room temperature
RWC: Radial water canal
SEM: Scanning electron microscopy
SM: Skeletogenic matrix
TEM: Transmission electron microscopy
TFs: Transcription factors
WMISH: Whole mount in situ hybridization

## References

[1] Brockes JP, Kumar A (2008). Comparative aspects of animal regeneration. Annu Rev Cell Dev Biol.

[2] Tanaka EM, Reddien PW (2011). Review the cellular basis for animal regeneration. Dev Cell.

[3] King RS, Newmark PA. The cell biology of regeneration. J Cell Biol. 2012;196(5):553–62. 10.1083/jcb.201105099 PMC3307701 22391035

[4] Slack JM (2017). Animal regeneration: ancestral character or evolutionary novelty?. EMBO Rep.

[5] Dupont S, Thorndyke M (2007). Bridging the regeneration gap: insights from echinoderm models. Nat Rev Genet.

[6] Ramon-mateu J, Ellison ST, Angelini TE, Martindale MQ (2019). Regeneration in the ctenophore *Mnemiopsis leidyi* occurs in the absence of a blastema, requires cell division, and is temporally separable from wound healing.

[7] Goss RJ (1992). The evolution of regeneration: adaptive or inherent?. J Theor Biol.

[8] Bely AE, Nyberg KG (2010). Evolution of animal regeneration: re-emergence of a field. Trends Ecol Evol.

[9] Vickery MCL, Vickery MS, Amsler CD, Mcclintock JB (2001). Regeneration in echinoderm larvae. Microsc Res Tech.

[10] Candia Carnevali MD, Burighel P. Regeneration in echinoderms and ascidians. eLS. 2010:1–15.

[11] Ben Khadra Y, Sugni M, Ferrario C, Bonasoro F, Oliveri P, Martinez P, Candia Carnevali MD (2018). Regeneration in stellate echinoderms: Crinoidea, Asteroidea, and Ophiuroidea. Results Probl Cell Differ.

[12] Cary GA, Wolff A, Zueva O, Pattinato J, Hinman VF (2019). Analysis of sea star larval regeneration reveals conserved processes of whole-body regeneration across the metazoa. BMC Biol.

[13] Candia Carnevali MD, Bonasoro F. Microscopic overview of crinoid regeneration. Microsc Res Tech. 2001;426:403–26. 10.1002/jemt.1187 11782071

[14] Dupont S, Thorndyke MC (2006). Growth or differentiation? Adaptive regeneration in the brittle star *Amphiura filiformis*. J Exp Biol.

[15] Weber J, Greer R, Voight B, White E, Roy R (1969). Unusual strength properties of echinoderm calcite related to structure. J Ultrasructure Res.

[16] Bottjer DJ, Davidson EH, Peterson KJ, Cameron RA (2006). Paleogenomics of echinoderms. Science..

[17] Märkel K, Röser U (1985). Comparative morphology of echinoderm calcified tissues: histology and ultrastructure of ophiuroid scales (Echinodermata, Ophiuroidea). Zoomorphology..

[18] Byrne M (1994). Ophiuroidea. Microscopic anatomy of invertebrates.

[19] Smith AB (1999). Biomineralization in echinoderms. Skelet Biominer Patterns Process Evol Trends.

[20] Wilt FH (2002). Biomineralization of the spicules of sea urchin embryos. Zool Sci.

[21] Wilt FH, Killian CE, Livingston BT (2003). Development of calcareous skeletal elements in invertebrates. Differ Rev.

[22] Matranga V, Pinsino A, Bonaventura R, Costa C, Karakostis K, Martino C, Russo R, Zito F (2013). Cellular and molecular bases of biomineralization in sea urchin embryos. Cah Biol Mar.

[23] Dylus DV, Czarkwiani A, Stångberg J, Ortega-Martinez O, Dupont S, Oliveri P (2016). Large-scale gene expression study in the ophiuroid *Amphiura filiformis* provides insights into evolution of gene regulatory networks. Evodevo..

[24] Dylus DV, Czarkwiani A, Blowes LM, Elphick MR, Oliveri P. Developmental transcriptomics of the brittle star *Amphiura filiformis* reveals gene regulatory network rewiring in echinoderm larval skeleton evolution. Genome Biol. 2018;19:26. 10.1186/s13059-018-1402-8 PMC5831733 29490679

[25] Czarkwiani A, Dylus DV, Carballo L, Oliveri P. FGF signalling plays similar roles in development and regeneration of the skeleton in the brittle star *Amphiura filiformis*. bioRxiv. 2019:632968. 10.1242/dev.180760 PMC8180256 34042967

[26] Oliveri P, Tu Q, Davidson EH (2008). Global regulatory logic for specification of an embryonic cell lineage. Proc Natl Acad Sci.

[27] Röttinger E, Saudemont A, Duboc V, Besnardeau L, Mcclay D, Lepage T, et al. FGF signals guide migration of mesenchymal cells, control skeletal morphogenesis of the skeleton and regulate gastrulation during sea urchin development. Development. 2008;785:353–65. 10.1242/dev.014282 18077587

[28] Rafiq K, Cheers MS, Ettensohn CA (2012). The genomic regulatory control of skeletal morphogenesis in the sea urchin. Development..

[29] Dubois P, Jangoux M (1990). Stereom morphogenesis and differentiation during regeneration of adambulacral spines of *Asterias rubens* (Echinodermata, Asteroida). Zoomorphology..

[30] Dubois P, Ameye L (2001). Regeneration of spines and pedicellariae in echinoderms: a review. Microsc Res Tech.

[31] Gorzelak P, Stolarski J, Dubois P, Kopp C, Meibom A (2011). 26Mg labeling of the sea urchin regenerating spine: insights into echinoderm biomineralization process. J Struct Biol.

[32] Heatfield BM, Travis DF (1975). Ultrastructural studies of regenerating spines of the sea urchin *Strongylocentrotus purpuratus* I. Cell types without spherules. J Morphol.

[33] Czarkwiani A, Dylus DV, Oliveri P (2013). Expression of skeletogenic genes during arm regeneration in the brittle star *Amphiura filiformis*. Gene Expr Patterns.

[34] Czarkwiani A, Ferrario C, Dylus DV, Sugni M, Oliveri P. Skeletal regeneration in the brittle star *Amphiura filiformis*. Front Zool. 2016;13:18. 10.1186/s12983-016-0149-x PMC4841056 27110269

[35] Burns G, Ortega-Martinez O, Thorndyke MC, Peck LS, Dupont S, Clark MS (2011). Dynamic gene expression profiles during arm regeneration in the brittle star *Amphiura filiformis*. J Exp Mar Biol Ecol.

[36] Biressi ACM, Zou T, Dupont S, Dahlberg C, Di Benedetto C, Bonasoro F (2010). Wound healing and arm regeneration in *Ophioderma longicaudum* and *Amphiura filiformis* (Ophiuroidea, Echinodermata): comparative morphogenesis and histogenesis. Zoomorphology..

[37] Delroisse J, Ortega-Martinez O, Dupont S, Mallefet J, Flammang P (2015). De novo transcriptome of the European brittle star *Amphiura filiformis* pluteus larvae. Mar Genomics.

[38] Agata K, Saito Y, Nakajima E (2007). Unifying principles of regeneration I: epimorphosis *versus* morphallaxis.

[39] Howard-Ashby M, Materna SC, Brown CT, Chen L, Cameron RA, Davidson EH (2006). Gene families encoding transcription factors expressed in early development of *Strongylocentrotus purpuratus*. Dev Biol.

[40] Wu S-Y, McClay DR (2007). The Snail repressor is required for PMC ingression in the sea urchin embryo. Development..

[41] Thiery JP, Acloque H, Huang RYJ, Nieto MA (2009). Epithelial-mesenchymal transitions in development and disease. Cell..

[42] Rho HK, McClay DR (2011). The control of foxN2/3 expression in sea urchin embryos and its function in the skeletogenic gene regulatory network. Development..

[43] Wallin J, Wilting J, Koseki H, Fritsch R, Christ B, Balling R (1994). The role of Pax-1 in axial skeleton development. Development..

[44] Nakashima K, Zhou X, Kunkel G, Zhang Z, Deng JM, Behringer RR (2002). The novel zinc finger-containing transcription factor Osterix is required for osteoblast differentiation and bone formation. Cell..

[45] Cattell M, Lai S, Cerny R, Medeiros DM. A new mechanistic scenario for the origin and evolution of vertebrate cartilage. PLoS One. 2011;6(7):e22474. 10.1371/journal.pone.0022474 PMC3142159 21799866

[46] Tang W, Li Y, Osimiri L, Zhang C (2011). Osteoblast-specific transcription factor osterix (Osx) is an upstream regulator of Satb2 during bone formation. J Biol Chem.

[47] Sánchez RS, Sánchez SS (2013). Characterization of pax1, pax9, and uncx sclerotomal genes during *Xenopus laevis* embryogenesis. Dev Dyn.

[48] Jandzik D, Garnett AT, Square TA, Cattell MV, Yu J-K, Medeiros DM (2014). Evolution of the new vertebrate head by co-option of an ancient chordate skeletal tissue. Nature..

[49] Rafiq K, Shashikant T, McManus CJ, Ettensohn CA (2014). Genome-wide analysis of the skeletogenic gene regulatory network of sea urchins. Development..

[50] Mann K, Poustka AJ, Mann M (2008). The sea urchin (*Strongylocentrotus purpuratus*) test and spine proteomes. Proteome Sci.

[51] Mann K, Wilt FH, Poustka AJ (2010). Proteomic analysis of sea urchin (*Strongylocentrotus purpuratus*) spicule matrix. Proteome Sci.

[52] Seaver RW, Livingston BT (2015). Examination of the skeletal proteome of the brittle star *Ophiocoma wendtii* reveals overall conservation of proteins but variation in spicule matrix proteins. Proteome Sci.

[53] Ferrario C, Czarkwiani A, Dylus DV, Piovani L, Candia Carnevali MD, Sugni M, Oliveri P. Extracellular matrix gene expression during arm regeneration in *Amphiura filiformis*. Cell Tissue Res. 2020;381(3):411-26. 10.1007/s00441-020-03201-0 32350640

[54] Ferrario C, Ben Khadra Y, Czarkwiani A, Zakrzewski A, Martinez P, Colombo G, Bonasoro F, Candia Carnevali MD, Oliveri P, Sugni M (2018). Fundamental aspects of arm repair phase in two echinoderm models. Dev Biol.

[55] Candia Carnevali MD, Bonasoro F, Lucca E, Thorndyke MC (1995). Pattern of cell proliferation in the early stages of arm regeneration in the feather star *Antedon mediterranea*. J Exp Zool.

[56] García-Arrarás JE, Valentín-Tirado G, Flores JE, Rosa RJ, Rivera-Cruz A, San Miguel-Ruiz JE, et al. Cell dedifferentiation and epithelial to mesenchymal transitions during intestinal regeneration in *H. glaberrima*. BMC Dev Biol. 2011;11:61. 10.1186/1471-213X-11-61 PMC3207902 22004330

[57] Bannister R, McGonnell IM, Graham A, Thorndyke MC, Beesley PW (2007). Coelomic expression of a novel bone morphogenetic protein in regenerating arms of the brittle star *Amphiura filiformis*. Dev Genes Evol.

[58] Märkel K, Röser U (1983). The spine tissues in the echinoid *Eucidaris tribuloides*. Zoomorphology..

[59] Gl D, Lennarz WJ (1988). Skeletogenesis in the sea urchin embryo. Development..

[60] Gliznutsa LA, Dautov SS (2005). Ultrastructural peculiarities of the embryogenesis of the brittle star *Amphipholis kochii* (Lutken, 1972). Russ J Mar Biol.

[61] Khor JM, Guerrero-Santoro J, Ettensohn CA (2019). Genome-wide identification of binding sites and gene targets of Alx1, a pivotal regulator of echinoderm skeletogenesis. Development.

[62] Ettensohn CA, Dey D (2017). KirrelL, a member of the Ig-domain superfamily of adhesion proteins, is essential for fusion of primary mesenchyme cells in the sea urchin embryo. Dev Biol.

[63] Ben Khadra Y, Ferrario C, Di Benedetto C, Said K, Bonasoro F, Candia Carnevali MD (2015). Re-growth, morphogenesis, and differentiation during starfish arm regeneration. Wound Repair Regen.

[64] Smith AB (1980). Stereom microstructure of the echinoid test. Paleontology..

[65] Kaneto S, Wada H (2011). Regeneration of amphioxus oral cirri and its skeletal rods: implications for the origin of the vertebrate skeleton. J Exp Zool Part B Mol Dev Evol.

[66] Somorjai IML, Somorjai RL, Garcia-Fernàndez J, Escrivà H (2012). Vertebrate-like regeneration in the invertebrate chordate amphioxus. Proc Natl Acad Sci.

[67] Ferretti P. Regeneration of vertebrate appendages. eLS. 2001.

[68] Murdock DJ, Donoghue PC (2011). Evolutionary origins of animal skeletal biomineralization. Cells Tissues Organs.

[69] Weiner S, Addadi L (2011). Crystallization pathways in biomineralization. Annu Rev Mater Res.

[70] Morgulis M, Gildor T, Roopin M, Sher N, Malik A, Lalzar M, Dines M, de-Leon SB, Khalaily L, de-Leon SB. Possible cooption of a VEGF-driven tubulogenesis program for biomineralization in echinoderms. Proc Natl Acad Sci 2019;116(25):12353–12362. 10.1073/pnas.1902126116 PMC6589685 31152134

[71] Sinha KM, Zhou X (2013). Genetic and molecular control of osterix in skeletal formation. J Cell Biochem.

[72] Wilkie IA (2001). Autotomy as a prelude to regeneration in echinoderms. Microsc Res Tech.

[73] Candia Carnevali MD, Bonasoro F, Roland E, Smith A, Campbell A (1995). Arm regeneration and pattern formation in crinoids. Echinoderm Research.

[74] Ettensohn CA, Illies MR, Oliveri P, De Jong DL (2003). Alx1, a member of the Cart1/Alx3/Alx4 subfamily of Paired-class homeodomain proteins, is an essential component of the gene network controlling skeletogenic fate specification in the sea urchin embryo. Development..

[75] Davidson EH, Rast JP, Oliveri P, Ransick A, Calestani C, Yuh CH, Minokawa T, Amore G, Hinman V, Arenas-Mena C, Otim O (2002). A provisional regulatory gene network for specification of endomesoderm in the sea urchin embryo. Dev Biol.

[76] Materna SC, Howard-Ashby M, Gray RF, Davidson EH (2006). The C2H2 zinc finger genes of *Strongylocentrotus purpuratus* and their expression in embryonic development. Dev Biol.

[77] Juliano CE, Voronina E, Stack C, Aldrich M, Cameron AR, Wessel GM (2006). Germ line determinants are not localized early in sea urchin development, but do accumulate in the small micromere lineage. Dev Biol.

[78] Andrikou C, Iovene E, Rizzo F, Oliveri P, Arnone MI (2013). Myogenesis in the sea urchin embryo: the molecular fingerprint of the myoblast precursors. Evodevo.

[79] Livingston BT, Killian CE, Wilt F, Cameron A, Landrum MJ, Ermolaeva O (2006). A genome-wide analysis of biomineralization-related proteins in the sea urchin *Strongylocentrotus purpuratus*. Dev Biol.

[80] Sun Z, Ettensohn CA (2014). Signal-dependent regulation of the sea urchin skeletogenic gene regulatory network. Gene Expr Patterns.

[81] Dhordain P, Dewitte F, Desbiens X, Stehelin D, Duterque-Coquillaud M (1995). Mesodermal expression of the chicken erg gene associated with precartilaginous condensation and cartilage differentiation. Mech Dev.

[82] Schuff M, Rössner A, Donow C, Knöchel W (2006). Temporal and spatial expression patterns of FoxN genes in *Xenopus laevis* embryos. Int J Dev Biol.

[83] Tremblay M, Sanchez-Ferras O, Bouchard M. Gata transcription factors in development and disease. Dev. 2018;145:dev164384. 10.1242/dev.164384 30348673

[84] Wagner EF (2002). Functions of AP1 (Fos/Jun) in bone development. Ann Rheum Dis.

[85] Jin E-J, Park KS, Kim D, Lee Y-S, Sonn JK, Jung JC (2010). TGF-β3 inhibits chondrogenesis by suppressing precartilage condensation through stimulation of N-cadherin shedding and reduction of cRREB-1 expression. Mol Cells.

[86] Nieto MA (2002). The snail superfamily of zinc-finger transcription factors. Nat Rev Mol Cell Biol.

[87] Yue R, Shen B, Morrison SJ (2016). Clec11a/osteolectin is an osteogenic growth factor that promotes the maintenance of the adult skeleton. Elife..

[88] Sitara D, Aliprantis AO (2010). Transcriptional regulation of bone and joint remodeling by NFAT. Immunol Rev.

[89] Srinivas BP, Woo J, Leong WY, Roy S (2007). A conserved molecular pathway mediates myoblast fusion in insects and vertebrates. Nat Genet.

[90] Bar-Shavit Z (2007). The osteoclast: a multinucleated, hematopoietic-origin, bone-resorbing osteoimmune cell. J Cell Biochem.

[91] Iwai K, Ishii M, Ohshima S, Miyatake K, Saeki Y (2007). Expression and function of transmembrane-4 superfamily (tetraspanin) proteins in osteoclasts: reciprocal roles of Tspan-5 and NET-6 during osteoclastogenesis. Allergol Int.

[92] Zhu X, Mahairas G, Illies M, Cameron RA, Davidson EH, Ettensohn CA (2001). A large-scale analysis of mRNAs expressed by primary mesenchyme cells of the sea urchin embryo. Development..

[93] Illies MR, Peeler MT, Dechtiaruk AM, Ettensohn CA (2002). Identification and developmental expression of new biomineralization proteins in the sea urchin *Strongylocentrotus purpuratus*. Dev Genes Evol.

[94] Adomako-Ankomah A, Ettensohn CA (2011). P58-A and P58-B: novel proteins that mediate skeletogenesis in the sea urchin embryo. Dev Biol.

[95] Geiss GK, Bumgarner RE, Birditt B, Dahl T, Dowidar N, Dunaway DL, Fell HP, Ferree S, George RD, Grogan T, James JJ, Maysuria M, Mitton JD, Oliveri P, Osborn JL, Peng T, Ratcliffe AL, Webster PJ, Davidson EH, Hood L, Dimitrov K (2008). Direct multiplexed measurement of gene expression with color-coded probe pairs. Nat Biotechnol..

