# Supplementary material for: Ultrastructural and molecular analysis of the origin and differentiation of cells mediating brittle star skeletal regeneration
Source: BMC Biol. 2021 Jan 18;19:9. doi: 10.1186/s12915-020-00937-7 (PMC7814545; doi:10.1186/s12915-020-00937-7)
Supplement: Supplementary file 1 — Additional file 1: Figure S1. Ultrastructural analysis of dermal cells at stage 4/5 of regeneration. A) TEM micrographs of a morula-like (or spherule) cell under the epidermis characterized by the presence of large inclusions, indicated by arrowhead. B) TEM micrograph of a phagocyte showing large nucleus with evident nucleolus, phagosome, indicated by arrowhead, and long cytoplasmic projections. Both cells have morphologies that resemble adult coelomocytes. Scale bars = 2 μm. EP = epidermis; RNC = radial nerve cord. Figure S2. Mesenchymal cells adjacent to the epidermis appear more differentiated proximally (closer to the amputation plane) then distally (at the tip) as well as in later stages of development (stages 4/5) compared to early ones (stages 2/3). Top left, frontal section schematics of a regenerating arm at stage 2/3 or 4/5. Top right, cross sections of stage 2/3 and 4/5 where letters in red squares indicate the position of TEM micrographs at the bottom. The area highlighted in blue/green is the dermal layer. A) Mesenchymal cells at stage 2/3 at the distal-most tip of the regenerate. Red arrow shows the presence of fibrils. B) Mesenchymal cells at stage 4/5 in the area right next to where ACC and RNC meet on either side of the RWC. C) Mesenchymal cells at stage 4/5 in the distal-most area of the regenerate in-between the ACC and the RWC. Red triangles indicate phagocytes, red circle indicates apoptotic cell, with characteristic nucleus. D) Mesenchymal cell at stage 4/5 in the area right next the epidermis (EP). Asterisk indicates cytoplasmic pocket. ACC = aboral coelomic cavity, RNC = radial nerve cord, RWC = radial water canal, St = stage, scale bars = 2 μm. Figure S3. Vertebral primordia at late stages of regeneration form between the ACC and the RWC. A) Semi-thin cross section of a late stage regenerating arm. B) Schematics of A. Purple= epidermis, Pink= RNC, Blue=RWC, Yellow= ACC, Grey= skeletal tissues. Figure S4. Ultrastructural analysis of different [file 12915_2020_937_MOESM1_ESM.docx]

**Additional file 1**


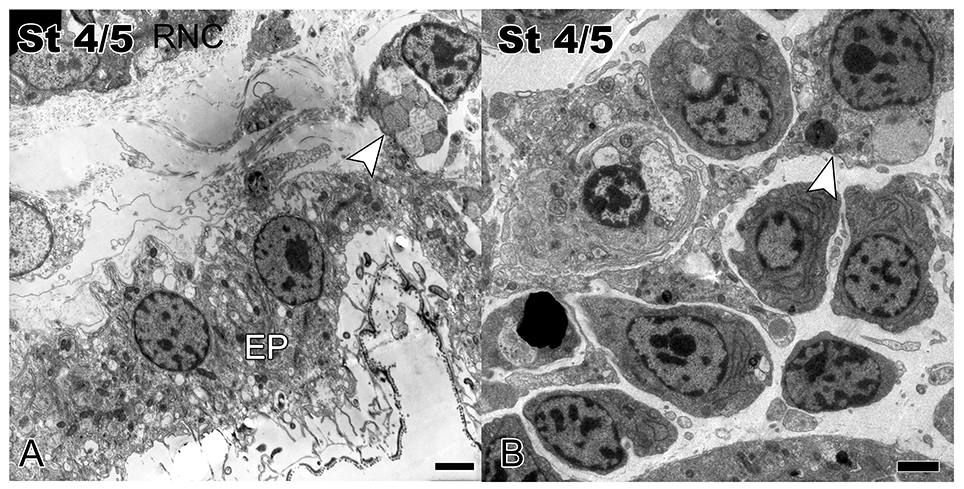


**Figure S1**: Ultrastructural analysis of dermal cells at stage 4/5 of regeneration. A) TEM micrographs of a morula-like (or spherule) cell under the epidermis characterized by the presence of large inclusions, indicated by arrowhead. B) TEM micrograph of a phagocyte showing large nucleus with evident nucleolus, phagosome, indicated by arrowhead, and long cytoplasmic projections. Both cells have morphologies that resemble adult coelomocytes. Scale bars = 2 µm. EP = epidermis; RNC = radial nerve cord.


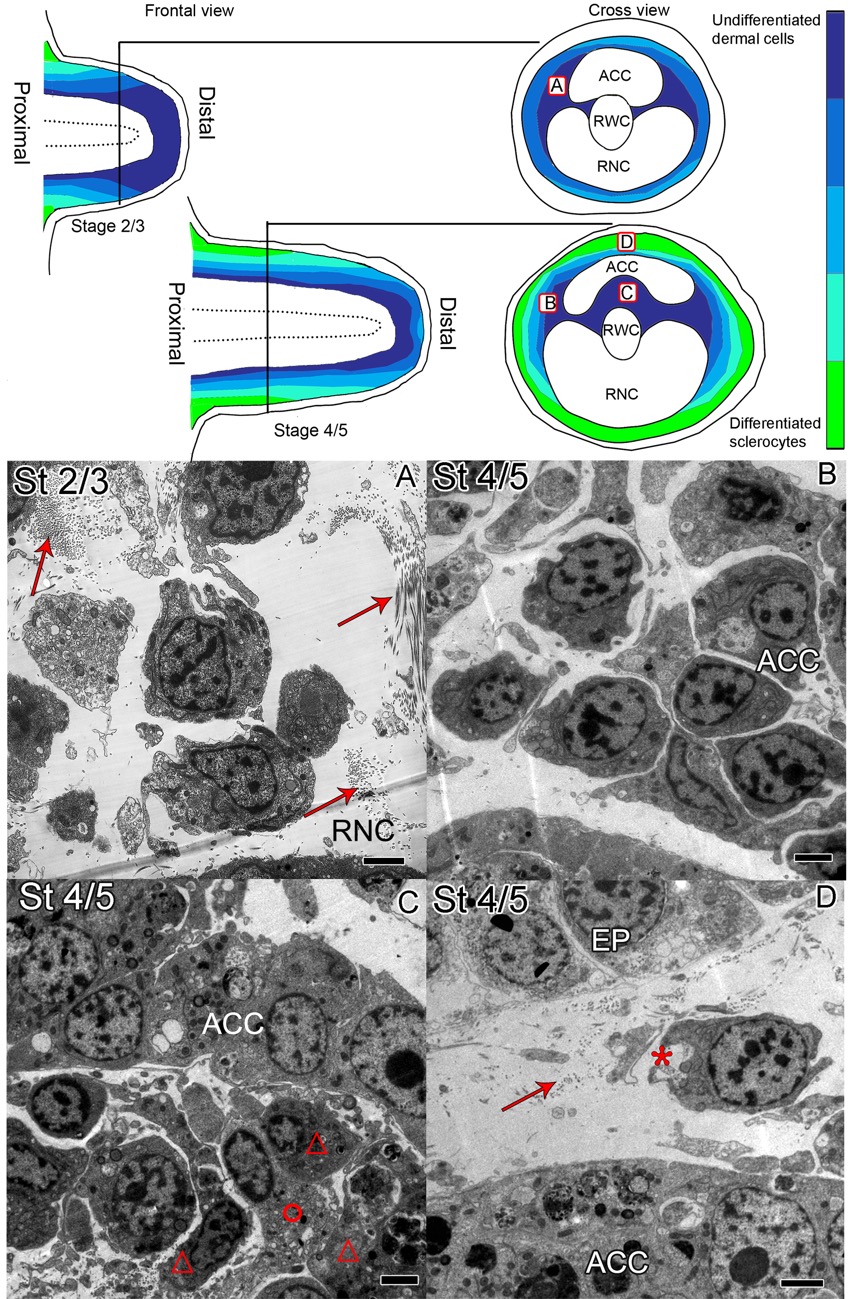


**Figure S2**: Mesenchymal cells adjacent to the epidermis appear more differentiated proximally (closer to the amputation plane) then distally (at the tip) as well as in later stages of development (stages 4/5) compared to early ones (stages 2/3). Top left, frontal section schematics of a regenerating arm at stage 2/3 or 4/5. Top right, cross sections of stage 2/3 and 4/5 where letters in red squares indicate the position of TEM micrographs at the bottom. The area highlighted in blue/green is the dermal layer. A) Mesenchymal cells at stage 2/3 at the distal-most tip of the regenerate. Red arrow shows the presence of fibrils. B) Mesenchymal cells at stage 4/5 in the area right next to where ACC and RNC meet on either side of the RWC. C) Mesenchymal cells at stage 4/5 in the distal-most area of the regenerate in-between the ACC and the RWC. Red triangles indicate phagocytes, red circle indicates apoptotic cell, with characteristic nucleus. D) Mesenchymal cell at stage 4/5 in the area right next the epidermis (EP). Asterisk indicates cytoplasmic pocket. ACC = aboral coelomic cavity, RNC = radial nerve cord, RWC = radial water canal, St = stage, scale bars = 2 µm.


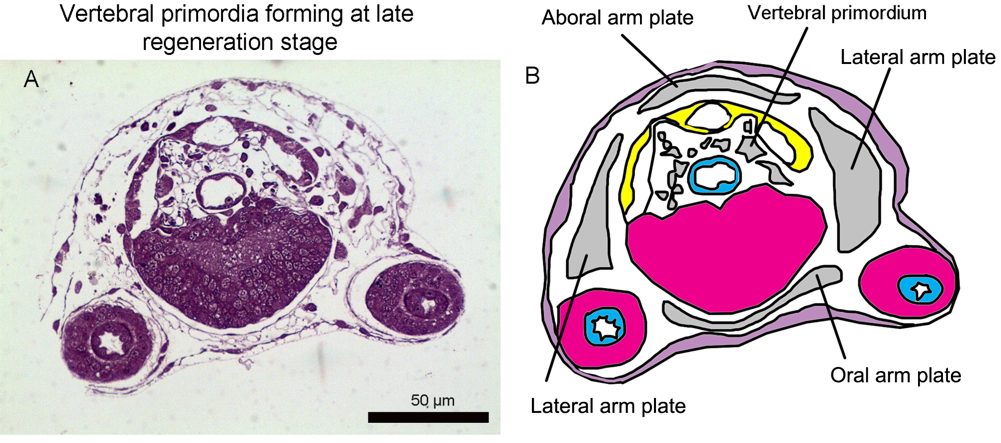


**Figure S3**: Vertebral primordia at late stages of regeneration form between the ACC and the RWC. A) Semithin cross section of a late stage regenerating arm. B) Schematics of A. Purple= epidermis, Pink= RNC, Blue=RWC, Yellow= ACC, Grey= skeletal tissues.

**
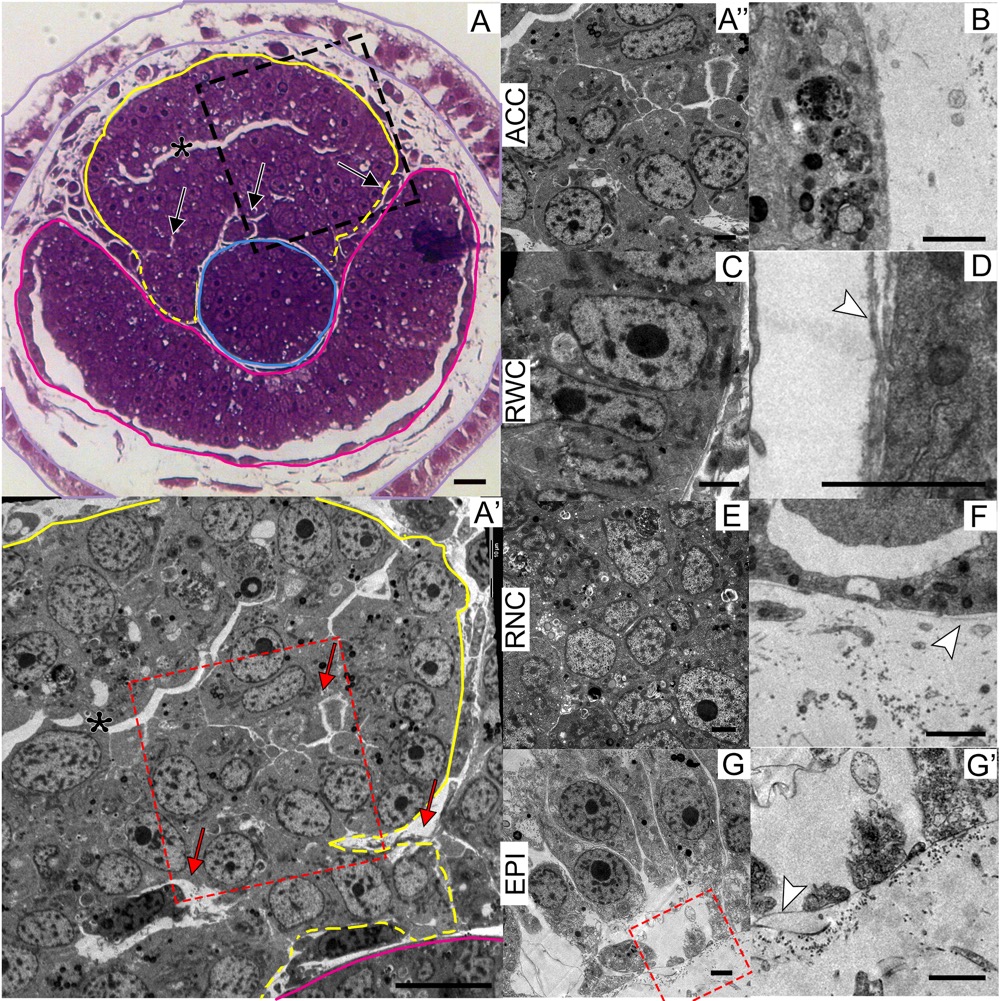
**

**Figure S4**: Ultrastructural analysis of different tissues of regenerating arms shows that the ACC is lacking a basal lamina and is less compact then other tissues of the regenerating arm. A) Semithin cross section of the tip of a regenerating arm. Coloured lines indicate the borders of the different structures (purple = epidermis, yellow = ACC, blue = RWC, pink = RNC). Black arrows indicate where cells of the ACC appear loosely connected. Yellow line is dotted where ACC borders are hard to distinguish. Black dotted square indicates approximate location of A’. Asterisk marks the ACC lumen. A’) TEM micrograph of the ACC, red arrows indicate where cells of the ACC appear loosely connected. Yellow line is dotted where ACC borders are hard to distinguish. Red dotted square indicates approximate location of A’’. B) aboral coelomic cavity (ACC); C and D) radial water canal (RWC); E and F) radial nerve cord (RNC); G epidermis (EPI); G’) is a magnification of G as indicated by the dotted red square. Arrowheads indicate basal lamina, when present. Scale bars of A and B are 10 µm, other scale bars are 2 µm.
