# Supplementary material for: Ultrastructural and molecular analysis of the origin and differentiation of cells mediating brittle star skeletal regeneration
Source: BMC Biol. 2021 Jan 18;19:9. doi: 10.1186/s12915-020-00937-7 (PMC7814545; doi:10.1186/s12915-020-00937-7)
Supplement: Supplementary file 2 — Additional file 2 : Figure S5. Details of spatial gene expression at early stages of regeneration show that many skeletogenic TFs are expressed in the ACC. Top of the figure shows schematics of sagittal, frontal and sections of early regenerating arm. First column shows schematic summary of gene expression as shown in Fig 4. For Afi-foxN2/3, Afi-twist and Afi-snail sections of WMISH are shown in sagittal and/or cross session. For Afi-soxE different focal planes in frontal view from aboral side are shown, while Afi-sp7/8 is imaged in semi-frontal view with focus on the regenerate tip. The arms are oriented aborally or orally for imaging and different focal planes inform on whether the tissue shown is the aboral epidermis, the ACC or RWC when imaged from the aboral side; or the oral epidermis, the RNC or the RWC when imaged from the oral side. All front views depicted here are imaged from the aboral side unless otherwise specified. Crossed arrows indicate the orientation of the section: A= aboral, O= oral, R= right, L= left, P= proximal, D= distal. Figure S6. Spatial gene expression at early and late stages of Afi-cara7la, Afi-rreb1, Afi-sp5, Afi-pax1/9 and Afi-foxN2/3. Columns on the left show the schematic of the arm in sagittal view for early stages; and in cross view for proximal and distal regenerates at late stages. Blue colour indicates the detected gene expression. Low-right images show the result of whole mount ISH of Afi-pax1/9 at two embryonic developmental stages, where the colorimetric staining is present in ectodermal cells. St. = stage. Figure S7. Levels of expression of genes in regenerating and non-regenerating arms. Graph shows the relative expression of skeletal genes in non-regenerating arms and at different stages of regeneration. Abundance of transcripts has been evaluated in 100 ng of total RNA using nCounter (Nanostring) technology. Relative expression (%) has been calculated using normalized counts per 100 ng of RNA relative to the maximum of [file 12915_2020_937_MOESM2_ESM.docx]

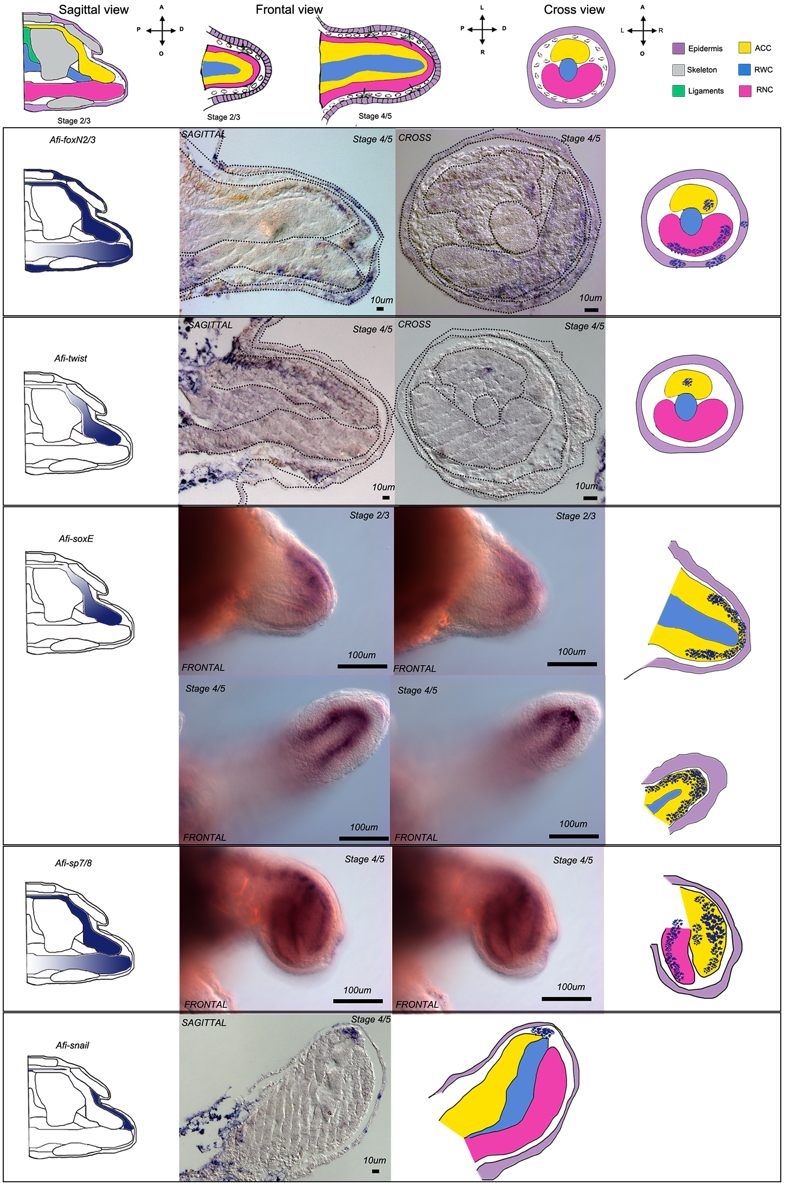


**Figure S5**: Details of spatial gene expression at early stages of regeneration show that many skeletogenic TFs are expressed in the ACC. Top of the figure shows schematics of sagittal, frontal and sections of early regenerating arm. First column shows schematic summary of gene expression as shown in Fig 4. For *Afi-foxN2/3, Afi-twist* and *Afi-snail* sections of WMISH are shown in sagittal and/or cross session. For *Afi-soxE* different focal planes in frontal view from aboral side are shown, while *Afi-sp7/8* is imaged in semi-frontal view with focus on the regenerate tip. The arms are oriented aborally or orally for imaging and different focal planes inform on whether the tissue shown is the aboral epidermis, the ACC or RWC when imaged from the aboral side; or the oral epidermis, the RNC or the RWC when imaged from the oral side. All front views depicted here are imaged from the aboral side unless otherwise specified. Crossed arrows indicate the orientation of the section: A= aboral, O= oral, R= right, L= left, P= proximal, D= distal.


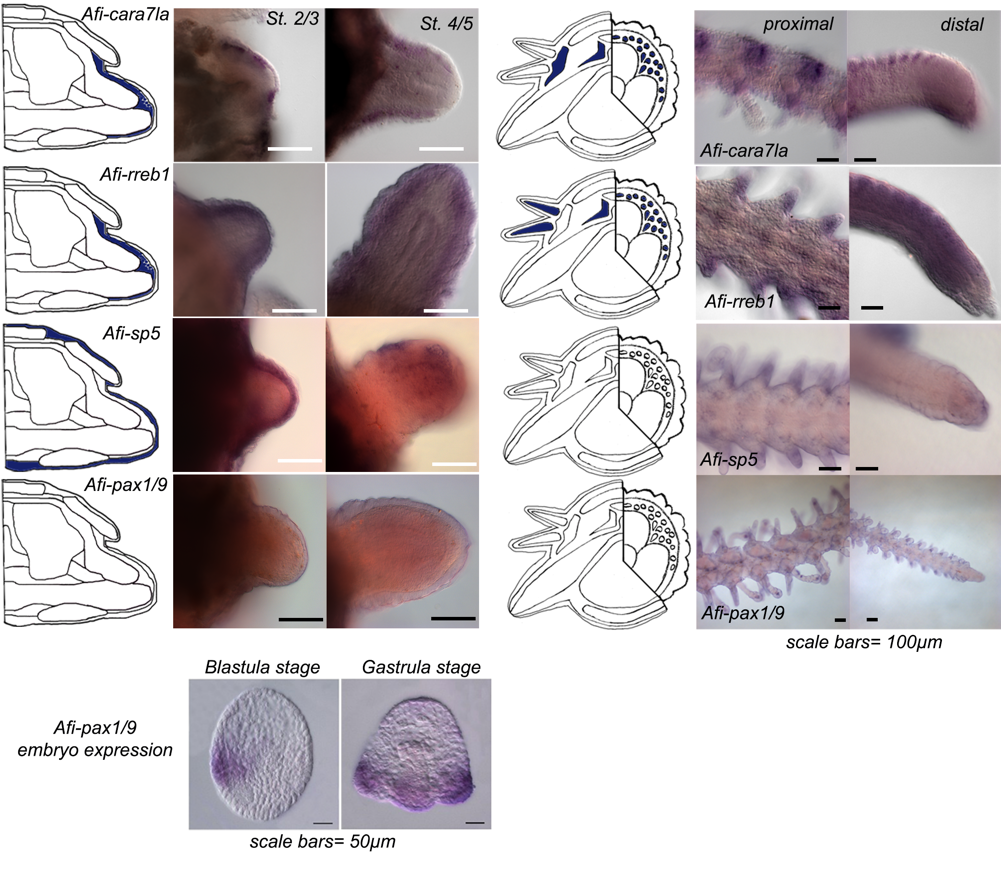


**Figure S6**: Spatial gene expression at early and late stages of *Afi-cara7la*, *Afi-rreb1*, *Afi-sp5*, *Afi-pax1/9 and Afi-foxN2/3*. Columns on the left show the schematic of the arm in sagittal view for early stages; and in cross view for proximal and distal regenrates at late stages. Blue colour indicates the detected gene expression. Low-right images show the result of whole mount ISH of *Afi-pax1/9* at two embryonic developmental stages, where the colorimetric staining is present in ectodermal cells. St. = stage.


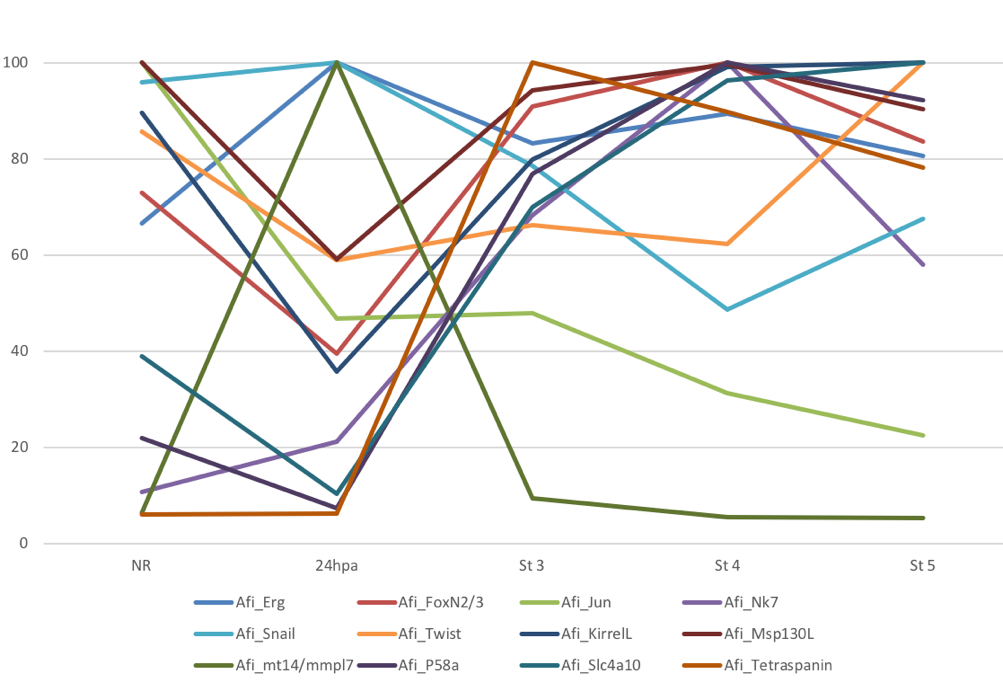


**Figure S7**: Levels of expression of genes in regenerating and non-regenerating arms. Graph shows the relative expression of skeletal genes in non-regenerating arms and at different stages of regeneration. Abundance of transcripts has been evaluated in 100 ng of total RNA using nCounter (Nanostring) technology. Relative expression (%) has been calculated using normalized counts per 100 ng of RNA relative to the maximum of expression for each gene. Non-regenerating arms (NR), 24 hours post amputation (24hpa), Stage 3 (St3), Stage 4 (St4) and Stage 5 (St5).


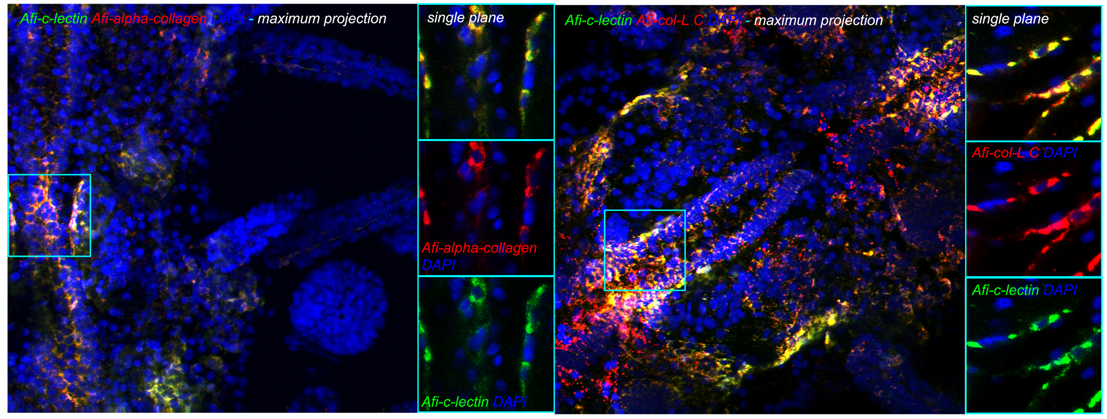


**Figure S8**: Fluorescent *in situ* hybridization showing co-localization of *Afi-c-lectin* with collagen genes in the skeletal elements. FISH of *Afi-c-lectin* (green) with collagen genes *Afi-alpha-collagen* (red - right) and *Afi-col-L C* (red - left) in regenerating arm at late stages. Large images are confocal maximal projections. Small images are enlargements of a single Z-stack slide and single fluorescent channel, as specified, with DAPI (blue) showing the coexpression of the two genes.


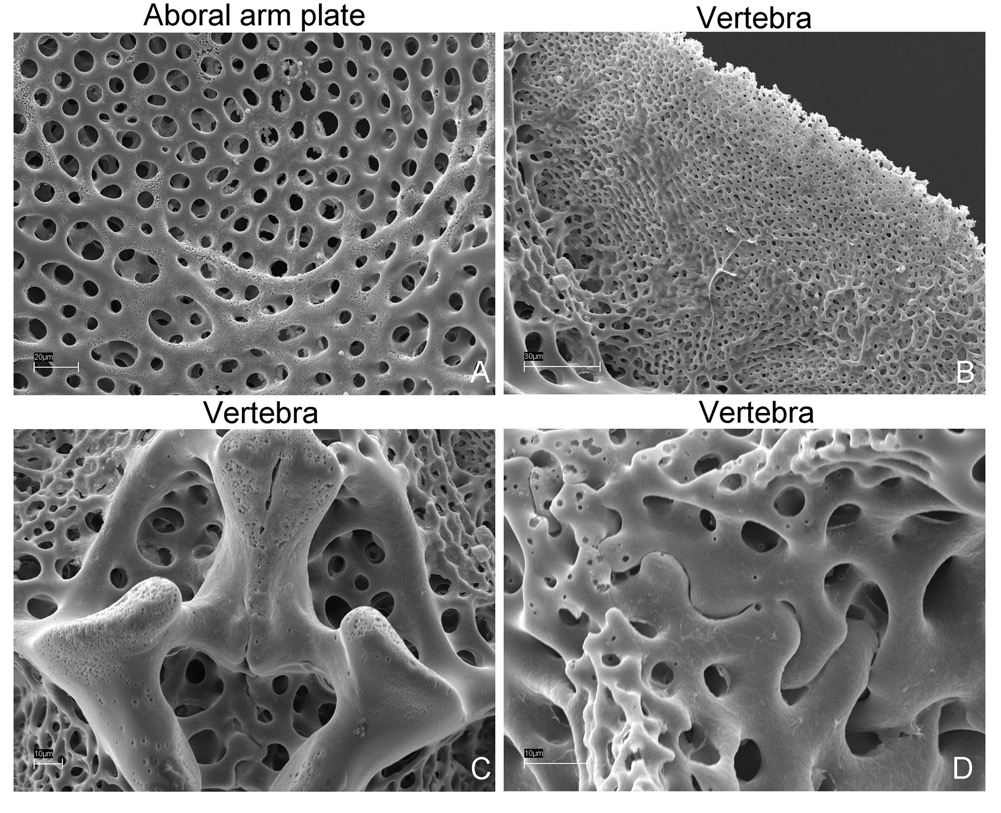


**Figure S9**: Different skeletal structures. SEM analysis in *A. filiformis* mature skeletal elements reveals different stereom structures and densities. A) Stereom of the aboral arm plate embedded in connective tissue shows large pores. B) Stereom of the vertebra from the point where the muscle attaches to it shows small pores. C) Compact stereom of the vertebral condylus. D) Median point where the left and the right vertebral halves fuse together during skeletal development. Scale bars: A = 20 µm; B = 30 µm; C and D = 10 µm.

**Table S1**: Levels of gene expression during regeneration. Nanostring normalised counts for skeletogenic genes in non-regenerating arm (NR), 24 hours post amputation (24hpa), 48 hours post amputation (48hpa), 72 hours post amputation (72hpa), Stage 3 (St3), Stage 4 (St4), Stage 5 (St5), proximal part of a 50% regenerated arm (50% prox) and distal part of a 50% regenerated arm (50% dist).

**Table S2**: Summary table and references of comparison of gene expression in the sea urchin *S. purpuratus* (Spu), in the brittle star *A. filiformis* (Afi) and in vertebrates (Vert). The data are used in the summary figure 6.

| ***Gene name in Afi*** | Afi | | Cit | Spu | Cit | Vert | Cit |  |  |
| --- | --- | --- | --- | --- | --- | --- | --- | --- | --- |
| ***alx1**** |  |  | 33 |  | 74 |  | 45 |  | Transcription factor |
| ***erg*** |  |  | 23 |  | 92 |  | 81 |  | skeletogenic |
| ***ets1/2**** |  |  | 33 |  | 26 |  | 45, 81 |  | non skeletogenic |
| ***foxN2/3*** |  |  |  |  | 26, 42 |  | 82 |  | ACC |
| ***gataC**** |  |  | 33, 23 |  | 75 |  | 83 | * | already published data |
| ***jun*** |  |  | 23 |  | 39 |  | 84 |  | no data available |
| ***nk7*** |  |  | 23 |  | 49 |  |  |  |  |
| ***pax1/9*** |  |  |  |  |  |  | 43, 47 |  |  |
| ***rreb**** |  |  | 25 |  | 76 |  | 85 |  |  |
| ***snail*** |  |  |  |  | 40 |  | 86, 41 |  |  |
| ***soxE*** |  |  |  |  | 77, 78 |  | 45, 48 |  |  |
| ***sp5*** |  |  |  |  |  |  | 44, 46 |  |  |
| ***sp7/8*** |  |  |  |  |  |  | 44, 47 |  |  |
| ***twist*** |  |  |  |  | 40 |  | 41, 45 |  |  |
| ***alpha-collagen**** |  |  | 33 |  | 79 |  | 45 |  |  |
| ***c-lectin**** |  |  | 23, 34 |  | 92 |  | 87 |  |  |
| ***cara7la**** |  |  | 24, 25 |  | 79 |  | 88 |  |  |
| ***kirrelL*** |  |  |  |  | 49 |  | 89 |  |  |
| ***msp130L*** |  |  | 24 |  | 93 |  |  |  |  |
| ***mt14/mmpl7*** |  |  | 24 |  | 51 |  | 90 |  |  |
| ***p19**** |  |  | 23, 34 |  | 93 |  |  |  |  |
| ***p58a*** |  |  | 23 |  | 94 |  |  |  |  |
| ***p58b**** |  |  | 23, 34 |  | 94 |  |  |  |  |
| ***scl4a10*** |  |  | 23 |  | 28 |  | 88 |  |  |
| ***tetraspanin*** |  |  | 23 |  | 92^ |  | 91 |  | ^ Sp_NET |

**Table S3**:

List of primers used to amplify and clone specific fragments of *A. filiformis* genes and to produce antisense probes for WMISH. F – forward primer, R – reverse primer, O – outer, I – inner, bp – base pair.

| **Primer Name** | **Forward/Reverse** | **primer sequence/clone ID** | **Fragment/probe length (bp)** | **Sequence Id** | **Publication/Database** |
| --- | --- | --- | --- | --- | --- |
| Afi-alpha-coll-F | F | Library clone P2A8 | 3000 | JG391435 | Burns et al, 2011 |
| Afi-alpha-coll-R | R | Library clone P2A8 | 3000 | JG391435 | Burns et al, 2011 |
| Afi-alx1-5O | 5' RACE-F | CTTGCGCCATTTAGCTCTG | 634 | KC788414 | Czarkwiani et al, 2013 |
| Afi-alx1-5I | 5' RACE-F | GCCATTTAGCTCTGCGATTT | 634 | KC788414 | Czarkwiani et al, 2013 |
| Afi-c-lectin-F | F | AGCAGCAATGAAGGTCTGGT | 1317 | KT936152 | Czarkwiani et al, 2016 |
| Afi-c-lectin-R | R | AAGACTGGAAGAAAACAAGA | 1317 | KT936152 | Czarkwiani et al, 2016 |
| Afi-cara7la-F | F | ACTTCTCTTTGGTCCGTCGA | 1207 |  | echinonet.eu |
| Afi-cara7la-R | R | TATAGCGGTACCTGCGTTGT | 1207 |  | echinonet.eu |
| Afi-col-L C-F | F | ACGTAAACGTTGGCATCTCC | 1014 | AfiCDS.id59066.tr822 | Ferrario et al, 2020 |
| Afi-col-L C-R | R | GTGATCGGCCTGATTGATCT | 1014 | AfiCDS.id59066.tr822 | Ferrario et al, 2020 |
| Afi-erg-F | F | GCGCATCGTGGTCAAATACC | 2149 | KM816844 | Dylus et al, 2016 |
| Afi-erg-R | R | GCTTGACGCAACTTGGGAAG | 2149 | KM816844 | Dylus et al, 2016 |
| Afi-foxN2/3-F | F | ATGCCACCCAATCGTAAGTC | 1590 | AfiCDS.id838.tr63418 | echinonet.eu |
| Afi-foxN2/3-R | R | TGCAGGTGATATTGCCTCTG | 1590 | AfiCDS.id838.tr63418 | echinonet.eu |
| Afi-jun-5O | 5' RACE-F | ACCATGGACGGATCAAACAT | 429 | KM816839 | Dylus et al, 2016 |
| Afi-jun-5I | 5' RACE-F | GCCATTTAGCTCTGCGATTT | 429 | KM816839 | Dylus et al, 2016 |
| Afi-kirrelL-F | F | GGTGAAACCGCAACTCTGAA | 1647 | id74191.tr58590 | echinonet.eu |
| Afi-kirrelL-R | R | TGTTGAGTTCGTATCTGCGC | 1647 | id74191.tr58590 | echinonet.eu |
| Afi-msp130L-F | F | CGTCTTACTCGTACCAGCCT | 878 | id75849.tr3754 | echinonet.eu |
| Afi-msp130L-R | R | CTACTCCTGCTGCTGTTCCT | 878 | id75849.tr3754 | echinonet.eu |
| Afi-nk7-F | F | TTCAGCCCGACAATGTTTCC | 1183 | id63655.tr58557 | echinonet.eu |
| Afi-nk7-R | R | CTTCGTCCCGCTTCCTCTT | 1183 | id63655.tr58557 | echinonet.eu |
| Afi-p58a-F | F | CCGTTCGAAACTAAGCATCGT | 600 | id59203.tr30563 | echinonet.eu |
| Afi-p58a-R | R | AGGTACCAGCTTTACTCTTGTT | 600 | id59203.tr30563 | echinonet.eu |
| Afi-pax1/9-F | F | CCGCAAGGCAAGATTTCAT | 1565 | afiReg.id382731.tr19736 | echinonet.eu |
| Afi-Pax1/9-1R | R | GGCGTCGCTTAACATAGACC | 1565 | afiReg.id382731.tr19736 | echinonet.eu |
| Afi-rreb1-F | F | TCAACTGCCAACGTCACATG | 893 | id64870.tr47807 | echinonet.eu |
| Afi-rreb1-R | R | CTTAGCTGCCGTCTGAGAGT | 893 | id64870.tr47807 | echinonet.eu |
| Afi-slc4a10-F | F | CGATCCCTACTCGGTTCCTC | 988 | id61902.tr45342 | echinonet.eu |
| Afi-slc4a10-R | R | TCGCAGTCTTCCATAGCGAT | 988 | id61902.tr45342 | echinonet.eu |
| Afi-soxE-F | F | TCACGACGACATGGAAAGAC | 1232 | id90635.tr47043 | echinonet.eu |
| Afi-soxE-R | R | GGACTGAATCTGCAACGTCC | 1232 | id90635.tr47043 | echinonet.eu |
| Afi-sp5-F | F | GCAACATGCCTGAACTGAAC | 1103 | afiReg.id146206.tr223106 | echinonet.eu |
| Afi-sp5-R | R | TTTCTTGACGGCATGTGTTT | 1103 | afiReg.id146206.tr223106 | echinonet.eu |
| Afi-sp7/8-F | F | CTGGACAGATTCCGCTTCTC | 794 | afiReg.id147919.tr368920 | echinonet.eu |
| Afi-Sp7/8-R | R | TACACGTTGGACACGCAAAT | 794 | afiReg.id147919.tr368920 | echinonet.eu |
| Afi-trspn-F | F | GGCGCTCGATGGCTGTTC | 716 | id30755.tr5407 | echinonet.eu |
| Afi-trspn-R | R | GAGGCTGTTTCCGTAAATCTTGA | 716 | id30755.tr5407 | echinonet.eu |
